# ETS factors are required but not sufficient for specific patterns of enhancer activity in different endothelial subtypes

**DOI:** 10.1016/j.ydbio.2021.01.002

**Published:** 2021-05

**Authors:** Alice Neal, Svanhild Nornes, Pakavarin Louphrasitthiphol, Natalia Sacilotto, Mark D. Preston, Lucija Fleisinger, Sophie Payne, Sarah De Val

**Affiliations:** a Department of Physiology, Anatomy and Genetics, University of Oxford, Oxford, OX1 3PT, United Kingdom; b Ludwig Institute for Cancer Research Ltd, Nuffield Department of Medicine, University of Oxford, Oxford, OX3 7DQ, United Kingdom; c National Institute for Biological Standards and Control, Blanche Lane, South Mimms, Potters Bar, EN6 3QG, United Kingdom

**Keywords:** Endothelial cell, Blood vessels, Arterio-venous differentiation, Artery, Vein, ETS, Transcription, Enhancer, Arterio-venous specification, ERG

## Abstract

Correct vascular differentiation requires distinct patterns of gene expression in different subtypes of endothelial cells. Members of the ETS transcription factor family are essential for the transcriptional activation of arterial and angiogenesis-specific gene regulatory elements, leading to the hypothesis that they play lineage-defining roles in arterial and angiogenic differentiation directly downstream of VEGFA signalling. However, an alternative explanation is that ETS binding at enhancers and promoters is a general requirement for activation of many endothelial genes regardless of expression pattern, with subtype-specificity provided by additional factors. Here we use analysis of *Ephb4* and *Coup-TFII* (*Nr2f2*) vein-specific enhancers to demonstrate that ETS factors are equally essential for vein, arterial and angiogenic-specific enhancer activity patterns. Further, we show that ETS factor binding at these vein-specific enhancers is enriched by VEGFA signalling, similar to that seen at arterial and angiogenic enhancers. However, while arterial and angiogenic enhancers can be activated by VEGFA *in vivo,* the *Ephb4* and *Coup-TFII* venous enhancers are not, suggesting that the specificity of VEGFA-induced arterial and angiogenic enhancer activity occurs via non-ETS transcription factors. These results support a model in which ETS factors are not the primary regulators of specific patterns of gene expression in different endothelial subtypes.

## Highlights

• Vein-specific enhancers can contain essential ETS motifs.
• VEGFA induced an increase in ETS binding at vein, arterial and angiogenic enhancers.
• VEGFA stimulation cannot induce vein-specific enhancer activity.

## 1 Introduction

The endothelial cell (EC) layer is the first part of the vascular system to form, initially via differentiation from progenitors (vasculogenesis), and later through the formation of new vessels from existing ones (angiogenesis). The vascular system is subdivided into arteries, veins, lymphatics and capillaries, each comprised of genetically distinct ECs expressing specific fate-determining genes (Lin et al., 2007; dela Paz and D’Amore, 2009). The essential balance of endothelial sprouting, proliferation and quiescence during angiogenesis also involves multiple genetically distinct EC subtypes (Potente et al., 2011; Rocha and Adams, 2009). However, while gene expression in the endothelium is known to involve dynamic transcriptional regulation, the signalling cascades and transcriptional effectors that establish and maintain these different endothelial cell fates have not been fully defined.

Complex spatiotemporal patterns of gene transcription during development are primarily regulated by a type of gene regulatory element known as enhancers. Enhancers, which can be located anywhere within a gene loci and sometimes beyond, are densely clustered groups of transcription factor motifs that bind an array of different transcription factors to activate transcription (Maston et al., 2006). In the endothelium, transcriptional regulation at gene enhancers is known to directly involve members of the ETS (E-26 transformation-specific) transcription factor family (De Val and Black, 2009). ETS proteins share a conserved DNA binding domain, binding DNA at a GGA(A/T) central motif which allows for much functional redundancy (Sharrocks, 2001). Multiple ETS transcription factors are expressed in the developing endothelium, and these have been implicated in numerous vascular processes (Randi et al., 2009). However, the precise role of ETS factors in regulating gene expression in the endothelium is unclear.

It has been hypothesised that vascular endothelial growth factor A (VEGFA) signalling may act via ETS transcription factors to enable specific activation of arterial and angiogenic genes. VEGFA signalling influences many processes during early vascular growth, and plays essential roles in vasculogenesis, arterial specification and angiogenesis (Olsson et al., 2006). ETS transcription factors are substrates of VEGFA-activated ERK signalling, and VEGFA-induced phosphorylation can increase ETS factor binding affinity (Yordy and Muise-Helmericks, 2000). The ability of high VEGFA levels to specifically activate components of the Notch signalling pathway in both arterial and angiogenic ECs has been directly linked to ETS factors: Decreased Notch pathway activity is observed after depletion of ERG, the most abundant ETS factor in mature ECs (Shah et al., 2017), and analysis of an intronic enhancer for the Notch ligand *Dll4* (termed Dll4in3 here) identified a group of ETS binding motifs required for arterial and angiogenic activity (Sacilotto et al., 2013; Wythe et al., 2013). ERG binding at the Dll4in3 enhancer increases with VEGFA stimulation and decreases with VEGFA inhibition (Fish et al., 2017; Wythe et al., 2013). A similar pattern of VEGFA-dependent ERG binding was also seen at the angiogenic EC-specific HLX-3 enhancer (Fish et al., 2017; Sacilotto et al., 2016), whilst VEGFA-induced ETS1 DNA binding and acetylation was linked to the increased RNAPII pause release at genes associated with angiogenesis (Chen et al., 2017).

Despite their hypothesised role in arterial and angiogenic-specific patterns of gene expression, binding motifs for ETS factors are also a common feature of many pan-endothelial expressed gene promoters and enhancers (De Val et al., 2008). ETS1, ERG and other ETS factors such as FLI1 are expressed throughout the endothelium, and ETS factors are known to be required for vasculogenesis and the establishment of endothelial identity (Birdsey et al., 2015; Casie Chetty et al., 2017; Lee et al., 2008). Further, a recent ChIP-seq study comparing cultured arterial and venous ECs found the ETS motif was over-represented at regions associated with both arterial-specific and vein-specific enhancer marks (Sissaoui et al., 2020). They also reported significant ERG binding peaks around venous gene loci, although these putative enhancer regions were not verified (Sissaoui et al., 2020). It is therefore still unclear whether ETS factors play a specific and lineage-defining role in the regulation of arterial and angiogenic patterns of gene expression downstream of VEGFA, or whether their role at the regulatory elements of these genes instead reflects a more general role for VEGFA-ETS in the endothelium.

In this paper, we undertake a detailed analysis of two recently characterized vein-enriched gene enhancers. We demonstrate that, similar to arterial and angiogenic enhancers, ETS factor binding at these venous enhancers is necessary for enhancer activation and vein-specific patterns of reporter gene expression, and that this binding is also enriched by VEGFA signalling. However, unlike arterial and angiogenic enhancers, these venous enhancers cannot be directly activated by over-expression of VEGFA *in vivo*. These results indicate that within the endothelium, VEGFA-stimulated ETS factor binding is a shared feature at enhancers associated with multiple different patterns of gene expression, and suggests that additional transcription factors may be primarily responsible for directing arterial, angiogenic and venous-specific gene expression patterns downstream of different growth factor signalling inputs.

## 2 Results

### 2.1 Vein EC-specific enhancers contain functional ETS binding motifs

We have recently identified enhancers within the venous-enriched *Ephb4* and *Coup-TFII* (*Nr2f2*) gene loci (Neal et al., 2019). Both enhancers (mouse DNA sequences termed Ephb4-2 and CoupTFII-965) drive robust reporter gene expression in venous ECs during arteriovenous specification in zebrafish and mouse transgenic models. In mice, the enhancers become progressively less active after embryonic stage (E)13 and silent in the adult (Neal et al., 2019; Payne et al., 2019). Ephb4-2 activity was specific to vein ECs, whilst CoupTFII-965 was also transiently active in the early dorsal aorta before E9.5, and in lymphatic ECs after mid-gestation similar to endogenous *Coup-TFII.* Neither enhancer was active in the mature microvasculature (Neal et al., 2019; Payne et al., 2019).

**Fig. 1:**
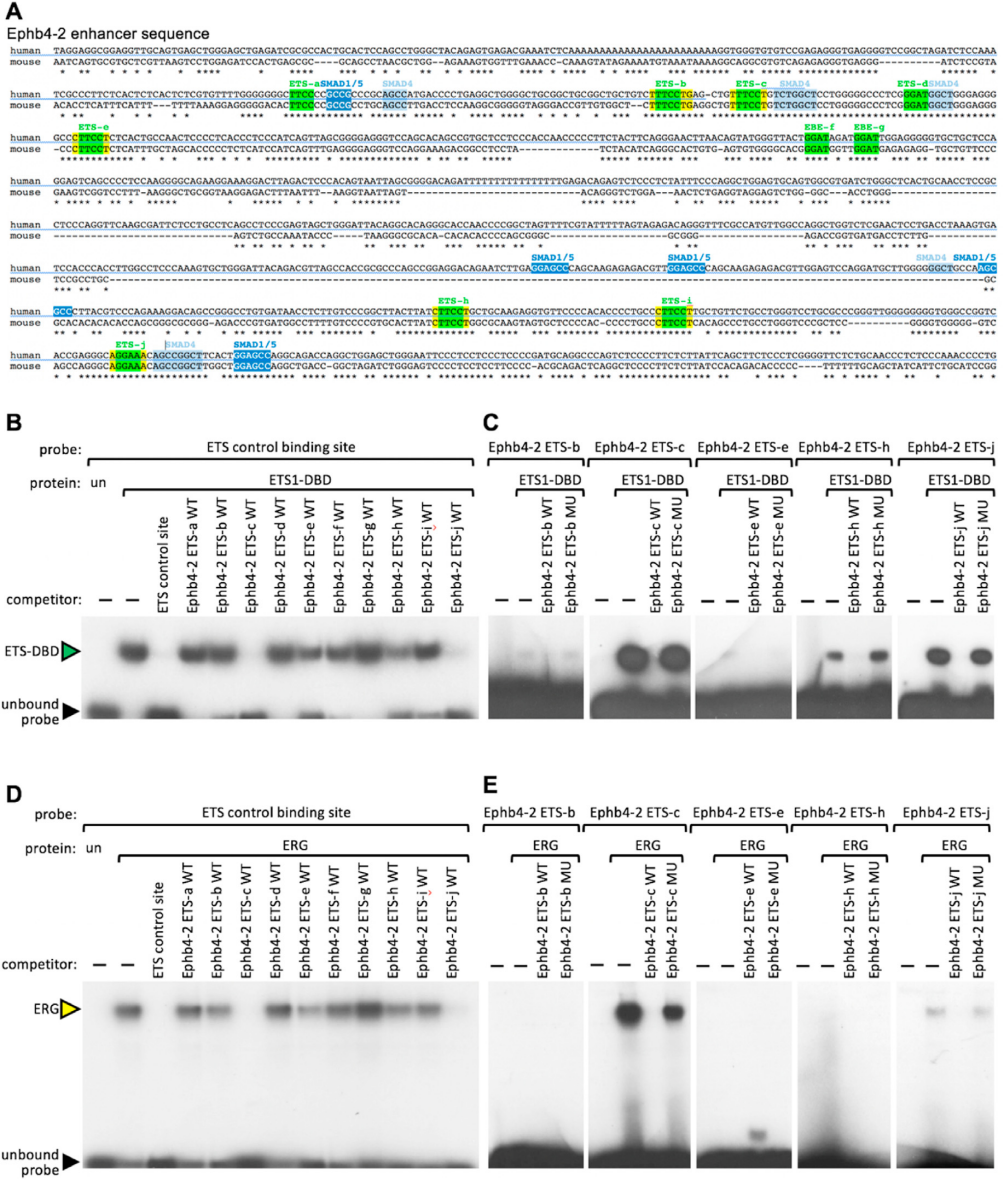
The Ephb4-2 venous enhancer contains functional ETS motifs. A. ClustalW alignment of the human and mouse sequences of the Ephb4-2 enhancer annotated with conserved ETS binding motifs (green), SMAD4 binding motifs (light blue) and SMAD1/5 binding motifs (dark blue) as previously reported (Neal et al., 2019). Flanking regions outside core ETS binding motifs which adhere to the ERG consensus motifs are indicated in yellow. ∗ denotes nt conserved between human and mouse sequences. B-E. Radiolabelled oligonucleotide probe encompassing a known ETS binding motif (ETS control consensus binding site, B and D) or putative Ephb4-2 ETS motif (ETS-b, ETS-c, ETS-e, ETS-h and ETS-j, C and E) was incubated with either unprogrammed TNT lysate (un), recombinant ETS1 DNA binding domain protein (ETS1-DBD, B–C) or ERG protein (D–E). Competitors added were either water control (−), an excess of unlabelled self-probe (ETS control site) or a single putative Ephb4-2 ETS wildtype (WT) or mutant (MU) motif. Gel shifts denoting protein binding are indicated by green (ETS-DBD) and yellow (ERG) arrowheads, unlabelled probe is indicated by black arrowhead.

Sequence analysis of the Ephb4-2 enhancer revealed ten core ETS binding motifs (GGA^A^/_T_) (Neal et al., 2019) and Fig. 1A). Of these, six motifs (termed Ephb4-2 ETS-b, -c, -e, -h, -i and -j) conformed to the canonical ERG binding motif ^A^/_C_GGAA^G^/_A_ (Kalna et al., 2019; Wei et al., 2010). We performed electrophoretic mobility shift assays (EMSAs) to define the ability of each putative ETS motif to bind a truncated ETS1 DNA binding domain protein (ETS1-DBD) (Fig. 1B–C) and full length ERG protein (Fig. 1D–E), both generated by *in vitro* transcription/translation. In competition with a radiolabelled control ETS motif, five ETS motifs within the Ephb4-2 enhancer (Ephb4-2 ETS-b, -c, -e, -h and -j) were able to compete for binding of either ETS1-DBD or ERG, or both proteins (Fig. 1B and D, with competition defined by decreased intensity of shifted band comparative to no-competitor lane). Of these, all were also able to directly bind ETS1-DBD to some degree (Fig. 1C) while Ephb4-2 ETS-c and -j were also able to directly bind ERG (Fig. 1E). The specificity of the protein motif interaction was clear as no shift was observed when the ETS motif was mutated for each site (Fig. 1C and E). Similar results were seen with the CoupTFII-965 enhancer. This enhancer contained eight human:mouse conserved core ETS motifs, three of which (Coup-965 ETS-b, -d and -g) conformed to the canonical ERG binding motif (Fig. S1A). Coup-965 ETS motifs ETS-d, -f and -g were able to compete with a radiolabelled control ETS motif for binding of ETS1-DBD and ERG, of which all were also able to directly bind ETS1-DBD to some degree. Further, ETS-d and ETS-g were also able to directly bind ERG (Fig. S1B-E). We concluded that, similar to arterial and angiogenically active enhancers, the Ephb4-2 and CoupTFII-965 venous-specific enhancers also contained multiple functional ETS motifs.

Since the binding of ETS factors to vascular enhancers has been previously associated with arterial-specific and angiogenic-specific enhancers, we next investigate whether ETS factor binding was also a feature at venous-specific enhancers (Fig. S2-4). We found significant binding for ERG, FLI1 and ETS1 at both Ephb4-2 and CoupTFII-965 venous enhancers (Fig. S2 using data from Chen et al., 2017; Nagai et al., 2018; Sissaoui et al., 2020). Interestingly, ERG binding peaks around these enhancers were seen in both human umbilical vein ECs (HUVECs) and human umbilical arterial ECs (HUAECs) (Fig. S2). Although arteriovenous identity in primary cell lines can be affected by extended passage in culture, the venous and arterial identity of these cells were confirmed prior to analysis and *EPHB4* and *COUP-TFII/NR2F2* were significantly enriched in these HUVECs (Sissaoui et al., 2020). Similar ERG, FLI1 and ETS1 binding peaks were found around the NRP2+26, MEF2F7 and EMCN-22 and EMCN-139 venous-enriched enhancers (Neal et al., 2019; Zhou et al., 2017). Again, ERG was bound in both HUVEC and HUAECs with the exception of EMCN-22, which had comparatively lower amounts of ERG binding in HUVECs and no detectable ERG bound in HUAECs (Fig. S2). As expected, enhancer-associated binding of ERG, FLI1 and ETS1 was not specific to these venous enhancers, as these datasets also show significant ETS binding at *in vivo* verified arterial, angiogenic and pan-endothelial enhancers (Fig. S3-4, focused on enhancers which have had their subtype-specific expression patterns previously validated in transgenic mouse models). Similar to the venous enhancers investigated, ERG binding around these arterial-, angiogenic- and pan-endothelial-expressed enhancers was seen in both HUVEC and HUAEC cells, suggesting that binding of ERG to specific enhancer regions was not routinely restricted to the EC subtypes in which the associated genes are preferentially active (Fig. S3). Taken together, these results demonstrate that functional ETS binding motifs can be present within vein EC-specific enhancers, and show that the ability to bind ERG and other ETS factors is not restricted to enhancers that are active in arterial and angiogenic ECs.

### 2.2 ETS motifs are required for activity of the Ephb4-2 and CoupTFII-965 venous enhancers

**Fig. 2:**
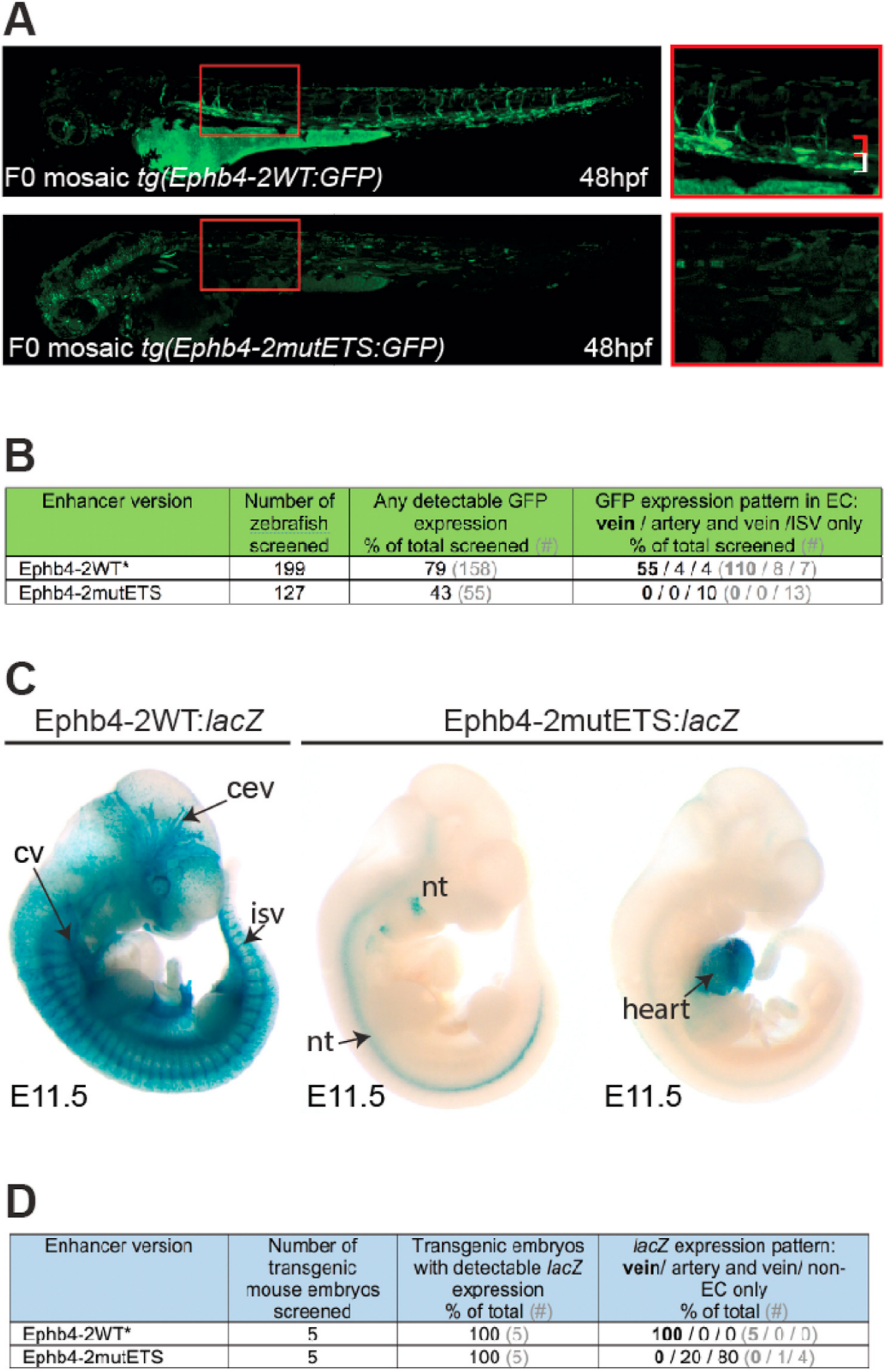
ETS factor motifs are required for venous Ephb4-2 enhancer activity. A. Representative 48hpf F0 Tol2-mediated mosaic transgenic zebrafish expressing either wild type (upper panel) or ETS-motif mutated (lower panel) versions of the Ephb4-2:GFP transgene. Red box denotes region shown at high magnification on the left, red bracket indicates dorsal aorta, white bracket indicates cardinal vein. B. Table summarizing the n numbers and patterns of GFP expression in F0 Tol2-mediated transgenic zebrafish. ∗ indicates transgenic zebrafish already reported in Neal et al. (2019). Note that the total numbers of zebrafish screened is lower than reported in Neal et al. (2019), as they exclude analysis that did not record vein/arterial/isv expression patterns. C. Representative E11.5 F0 transgenic mouse embryos expressing either wild type (left panel) or ETS-motif mutated (right panels) versions of Ephb4-2:lacZ transgenes. cev ​= ​branches of cerebral venous plexus, cv ​= ​cardinal vein, isv ​= ​intersomitic vessel, nt ​= ​neural tube. All additional transgenic embryos are shown in Fig. S5. D. Table summarizing the n numbers and patterns of X-gal staining in F0 transgenic mouse embryos. ∗ denotes data initially reported in Neal et al. (2019).

In agreement with previous *in vitro* studies (Sissaoui et al., 2020), our results demonstrate ETS factor binding at *in vivo*-validated venous enhancers. However, it has yet to be determined if ETS factors are required for endothelial activity of vein-specific enhancers. To clarify this we first generated a mutant version of the Ephb4-2 enhancer, in which each core binding region of EMSA-verified ETS motifs was mutated from GGA to TCA creating Ephb4-2mutETS (mutant ETS-b, -c, -e, -h and -j). EMSA analysis confirmed that the mutated ETS binding motifs could not bind ETS proteins (Fig. 1C and E). The Ephb4-2mutETS enhancer was cloned upstream of the E1b silent promoter and the GFP reporter gene for analysis in transgenic zebrafish, and upstream of the *hsp68* silent minimal promoter and *lacZ* reporter gene for analysis in transgenic mice. As previously reported, the Ephb4-2 enhancer was able to drive vein-enriched GFP reporter gene expression in mosaic F0 transgenic zebrafish at 48 ​h post fertilization (hpf) (Neal et al., 2019) and Fig. 2A–B). However, the modified Ephb4-2mutETS enhancer drove little reporter gene expression in ECs in transgenic zebrafish: fewer injected embryos expressed GFP, and this was predominantly in non-EC cells (Fig. 2A–B). We saw similar results in F0 transgenic mice. While the Ephb4-2WT enhancer directs *lacZ* expression (measured by blue X-gal staining) exclusively to the venous endothelium (Neal et al., 2019) and Fig. 2C–D), the Ephb4-2mutETS enhancer was not able to drive reporter gene activity in venous endothelial cells (Fig. 2C–D and Fig. S5). Only a single Ephb4-2mutETS:*lacZ* transgenic embryo showed any EC activity (Fig. 2D and Fig. S5), and this was in an expression pattern entirely different to Ephb4-2WT, suggesting it may have been influenced by transgene insertion location. All other transgenic embryos showed no activity in the vasculature, although some ectopic neural and cardiac activity was sporadically detected (Fig. 2C and Fig. S5). Similar results were also found with the CoupTFII-965 enhancer: The wildtype CoupTFII-965 enhancer was primarily active in venous ECs in both transgenic zebrafish and transgenic mice (Neal et al., 2019 and Fig. S6). However, the mutant CoupTFII-965mutETS (in which ETS-d, -f and -g were mutated) was unable to drive endothelial GFP expression in mosaic F0 transgenic zebrafish (Fig. S6A-B) and unable to drive vascular *lacZ* expression in F0 transgenic mice (Fig. S6C-D).

The loss of Ephb4-2 and CoupTFII-965 vein enhancer activity after ETS motif mutation observed here is similar to the loss of activity seen when ETS motifs are mutated in the arterial and angiogenic Dll4in3 enhancer (Sacilotto et al., 2013; Wythe et al., 2013), in other characterized arterial and angiogenic enhancers (Becker et al., 2016; Chiang et al., 2017; Robinson et al., 2014) and in pan-EC enhancers (De Val et al., 2004; Kappel et al., 2000; Prandini et al., 2005). Consequently, these results indicate that a requirement for ETS motifs is shared by EC-expressed gene enhancers with many different patterns of expression within the endothelium.

### 2.3 Reduction in ETS factor levels can result in reduced vein enhancer activity

The requirement for functional ETS motifs within vein-specific enhancers suggests that ETS transcription factors may be required to drive venous enhancer activity. To assess this directly, we next measured the activity of the vein-specific Ephb4-2 enhancer in stable transgenic zebrafish lines after morpholino knockdown of the ETS transcription factors *erg* and *fli1*. There are multiple ETS factors expressed in the developing zebrafish vasculature (Pham et al., 2007), and ETS binding motifs in venous enhancers can be occupied by multiple different ETS factors (Fig. S2). However, the reduction in endothelial ETS factor levels achieved by knockdown of the abundantly expressed *erg* and paralogue *fli1* will allow us to observe if a vein-specific enhancer is sensitive to changes in ETS factor signalling levels without ablating vasculogenesis and early endothelial differentiation. Additionally, since ERG has previously been linked to arterial and angiogenic gene activation and *erg/fli1* knockdown reduced Dll4 transgene activity (Wythe et al., 2013), this analysis also allows us to determine if ERG depletion specifically affects arterial and angiogenic enhancers or can have a similar effect on vein-specific enhancers.

**Fig. 3:**
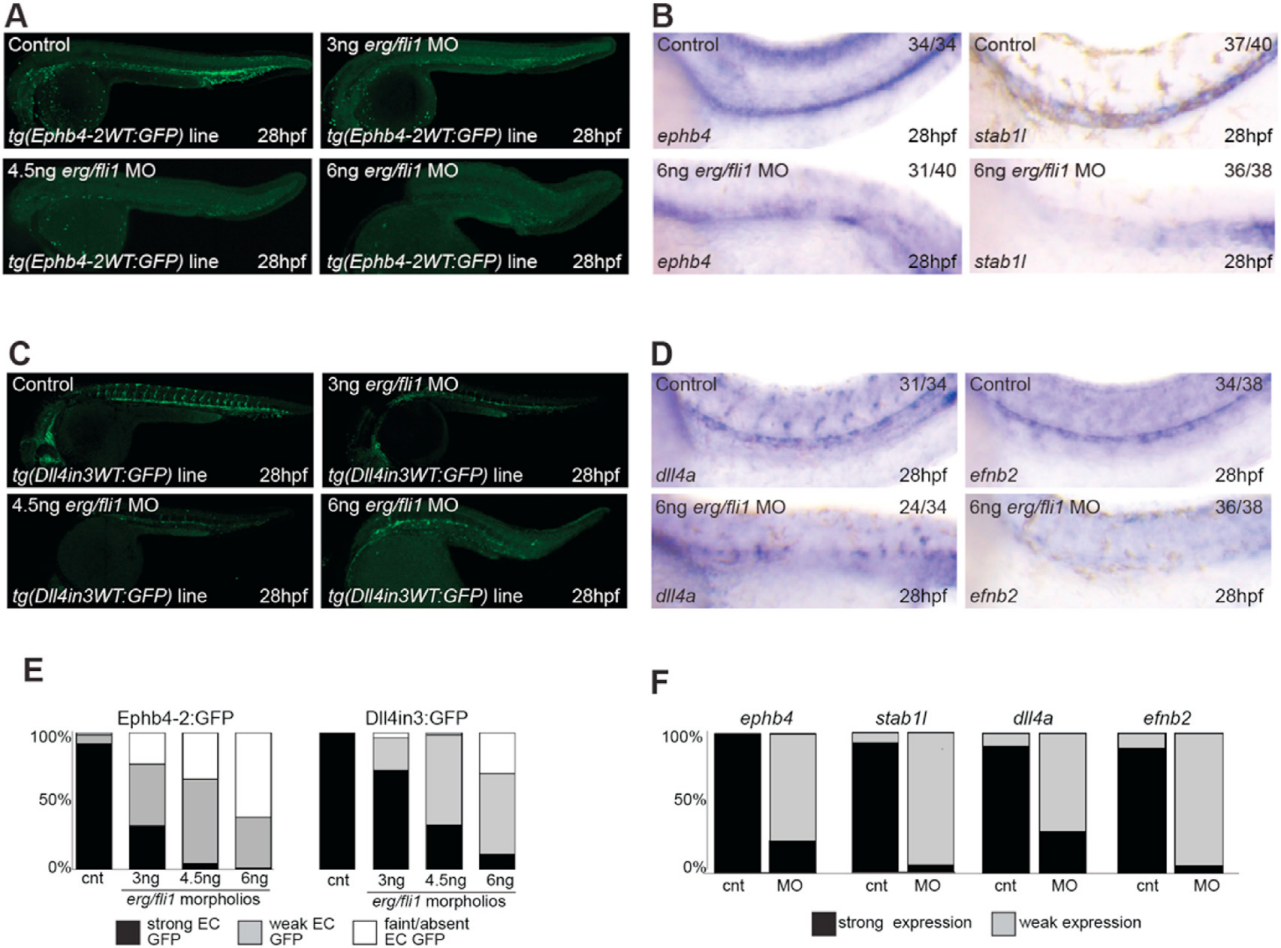
Knockdown of *erg* and *fli1* in zebrafish reduces activity of both venous and arterial enhancers, and reduces the endogenous expression of both venous and arterial genes. A-B. Representative *tg(Ephb4-2WT:GFP)* transgenic zebrafish (A), and wildtype zebrafish after whole-mount *in situ* hybridization for venous markers *ephb4* and *stab1l* (B) after morpholino-induced *erg/fli1* knockdown. C-D. Representative *tg(Dll4in3WT:GFP)* transgenic zebrafish (C), and wildtype zebrafish after whole-mount *in situ* hybridization for arterial markers *dll4a* and *efnb2* (D) after morpholino-induced *erg/fli1* knockdown. Numbers on top right of B and D indicate number of embryos with the predominant and displayed phenotype per total number of embryos analyzed. E-F. Graphs depicting observed GFP/endogenous gene expression levels. Ephb4-2:GFP cnt n ​= ​460, 3 ​ng MO n ​= ​420, 4.5 ​ng MO n ​= ​279, 6 ​ng MO n ​= ​341. Dll4in3:GFP cnt n ​= ​34, 3 ​ng MO n ​= ​29, 4.5 ​ng MO n ​= ​67, 6 ​ng MO n ​= ​27. *ephb4* cnt n ​= ​34, 6 ​ng MO n ​= ​40; *stab1l* cnt n ​= ​40, 6 ​ng MO n ​= ​38; *dll4a* cnt n ​= ​34, 6 ​ng MO n ​= ​34; *efnb2* cnt n ​= ​38, 6 ​ng MO n ​= ​38.

GFP expression in *tg(Ephb4-2WT:GFP)* zebrafish was significantly reduced after morpholino knockdown of *erg* and *fli1*, with the strength of Ephb4-2:GFP transgene activity inversely correlated with the levels of *erg/fli1* MO (Fig. 3A and E). While the vasculature was significantly phenotypically altered by *erg/fli1* knockdown, *kdr* and *kdrl* expression appeared unaffected by this depletion (Fig. S7A-B). Conversely, we saw a reduction in the expression of endogenous *ephb4* and *stab1l*, a zebrafish venous marker, when assessed by whole mount *in situ* hybridization analysis (Fig. 3B and F). High expression of *ephb4* outside of the vasculature meant the decrease of endothelial *ephb4* could not be reliably quantified by qRT-PCR. Although the enhancers regulating *stab1l* vein expression in zebrafish are not well defined, its locus contains two human-zebrafish conserved putative enhancer regions that bind ETS, ERG and SMAD1/5 in HUVECs, suggesting a similar mode of regulation to *Ephb4* (Fig. S7C). Similar reductions in the expression of some venous-enriched genes was also observed after ERG depletion in HUVECs (Sissaoui et al., 2020). Some reduction in GFP expression after *erg/fli1* knockdown was also observed in *tg(CoupTFII-*965WT*:GFP)* transgenic zebrafish, although this was not as marked (Fig. S8). As previously reported, *erg/fli1* knockdown also resulted in reduced activity of the arterial/angiogenic Dll4in3 enhancer in transgenic zebrafish (Fig. 3C and E), and in reduced expression of endogenous *dll4a* and *efnb2*, a zebrafish arterial marker (Fig. 3D and F). These results therefore indicate that expression of venous-specific enhancers and endogenous genes can be reduced by perturbations of ETS factor signalling in a similar manner to that of arterial and angiogenic enhancers.

### 2.4 ETS binding at vein EC-specific enhancers can increase after VEGFA stimulation

It has been previously proposed that VEGFA/ERK induced phosphorylation and activation of the ETS transcription factor ERG results in the specific induction of arterial and angiogenic-specific genes through increased binding to enhancer regions, as exemplified at the Dll4in3 and HLX-3 enhancers (Fish et al., 2017; Wythe et al., 2013). High VEGFA levels are a known inducer of arterial differentiation and angiogenesis (Red-Horse and Siekmann, 2019; Siekmann et al., 2008; Swift and Weinstein, 2009). Conversely, veins are exposed to lower VEGFA levels and venous EC-specific genes are not induced by VEGFA stimulation *in vitro* (Lawson et al., 2002; Rivera et al., 2011). Therefore, if VEGA-induced binding of ETS factors such as ERG at enhancer elements is responsible for arterial- and angiogenic-specific gene expression, a possible mode of action to achieve this specificity would be for this to not occur at venous enhancers. In this model, it would be expected that ETS binding at the Ephb4-2 and CoupTFII-965 venous enhancers would not increase after VEGFA stimulation. We therefore investigated whether ETS factor binding at these venous enhancers was sensitive to VEGFA signalling.

**Fig. 4:**
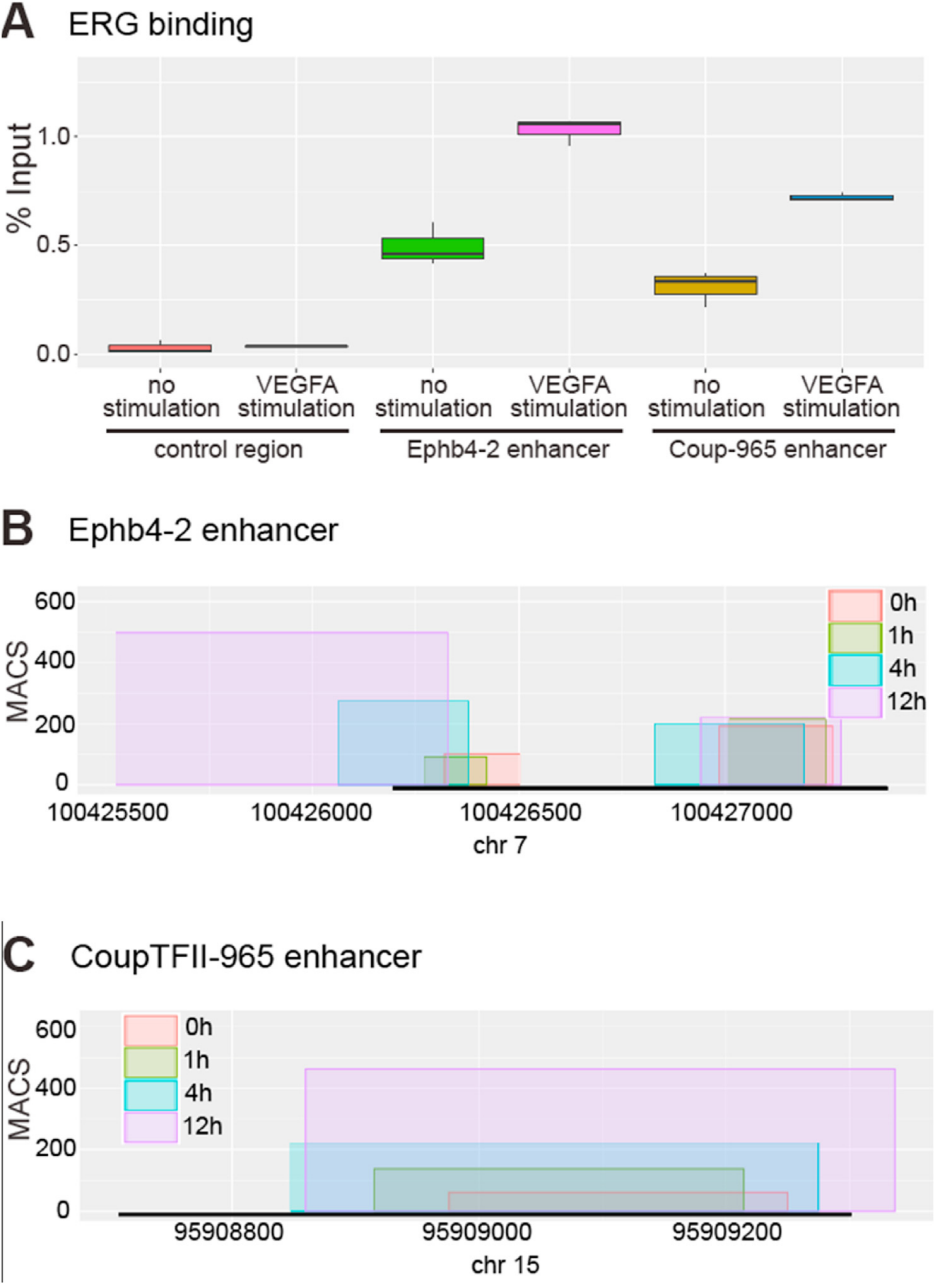
VEGFA signalling increases ETS factors binding to venous enhancers. A. HUVEC ERG binding ChIP-qPCR box-and-whiskers plot. ERG binding in unstimulated HUVECs is significantly enriched at the Ephb4-2 p ​< ​0.001 (green) and CoupTFII-965 p ​< ​0.001 (yellow) enhancers compared to the control region. Stimulation of HUVECs with VEGFA for 1.5 ​h prior to analysis resulted in significantly enriched ERG binding at both the Ephb4-2 p ​< ​0.001 (pink) and CoupTFII-965 p ​< ​0.001 (blue) enhancer regions compared to unstimulated conditions. No enrichment is observed between control regions (p ​= ​1.000). The six conditions show significant differences (ANOVA f-test, p ​< ​1 ​× ​10-9). Horizontal lines ​= ​medians, boxes ​= ​interquartile range (IQR); vertical lines ​= ​minimal/maximal values. Data represents three biological replicates each with three technical replicates performed in triplicate. All data points were included in statistical analysis. Figure S9 shows the data presented alongside the IgG controls. B–C. ETS1 binding at venous Ephb4-2 (B) and CoupTFII-965 (C) enhancer regions is increased in the hours after VEGFA stimulation. Box width indicates region of ETS1 binding and box height indicates the maximal MACS score for this region after 0h (red), 1 ​h (green), 4 ​h (blue) and 12 ​h (purple) of VEGFA stimulation. Black bar indicates orthologous enhancer region and x axis covers a 5 ​kb genomic region. Numbers indicate distance from transcriptional start site (TSS) of the Ephb4 (B) or Coup-TFII (C) gene. Data reanalysed from ETS1 ChIP-seq by Chen et al., (2017). Fig. S10 shows the data for other enhancers.

This analysis was performed in early passage HUVECs, the same cell line used to demonstrate venous-defining SMAD1/5 binding to the Ephb4-2 and CoupTFII-965 enhancers (Neal et al., 2019). We performed ChIP qPCR to examine ERG binding at the Ephb4-2 and CoupTFII-965 enhancers in HUVECs before and after VEGFA stimulation. In agreement with our EMSA analysis and previous ChIP-seq analysis (Fig. 1, Fig. S2 and Chen et al., 2017; Nagai et al., 2018; Sissaoui et al., 2020), we found statistically significant enrichment of ERG binding at both the Ephb4-2 and the CoupTFII-965 enhancer regions in HUVECs (Fig. 4A and Fig. S9). Strikingly, ERG binding at both enhancer regions was significantly increased after HUVECs were stimulated with VEGFA even though VEGFA-stimulation is not associated with venous gene expression (Fig. 4A and S9). In order to determine if ETS factor occupancy of venous enhancers and its concordant increase after VEGFA stimulation is specific to ERG, we next re-examined published ETS1 ChIP-seq data from serum starved or VEGFA-stimulated HUVECs (Chen et al., 2017). Similar to the binding of ERG at these regions, there was significant ETS1 binding to Ephb4-2 and CoupTFII-965 enhancer regions in all conditions, with an increase in peak size after VEGFA stimulation at both the Ephb4-2 and CoupTFII-965 enhancer regions (Fig. 4B and C). ETS1 binding increased with longer VEGFA stimulation time, with the highest peak seen after 12 ​h of VEGFA stimulation. Analysis of ETS1 binding patterns at the arterial-specific Ece1 intronic enhancer (Robinson et al., 2014), arterial and angiogenic Dll4in3 enhancer, and angiogenic HLX-3 enhancer demonstrated similar patterns of VEGFA-induced increased ETS1 binding (Fig. S10). Together, these results show that increased ETS factor binding after VEGFA stimulation at sub-type specific enhancers is not restricted to ERG. Further, as VEGFA-associated increase in ETS1 binding is seen at venous enhancers as well as at arterial and angiogenic enhancers, it is unlikely to directly account for the specific expression patterns of these enhancers within the endothelium.

### 2.5 Venous enhancer activity can be sensitive to changes in VEGFA signalling

**Fig. 5:**
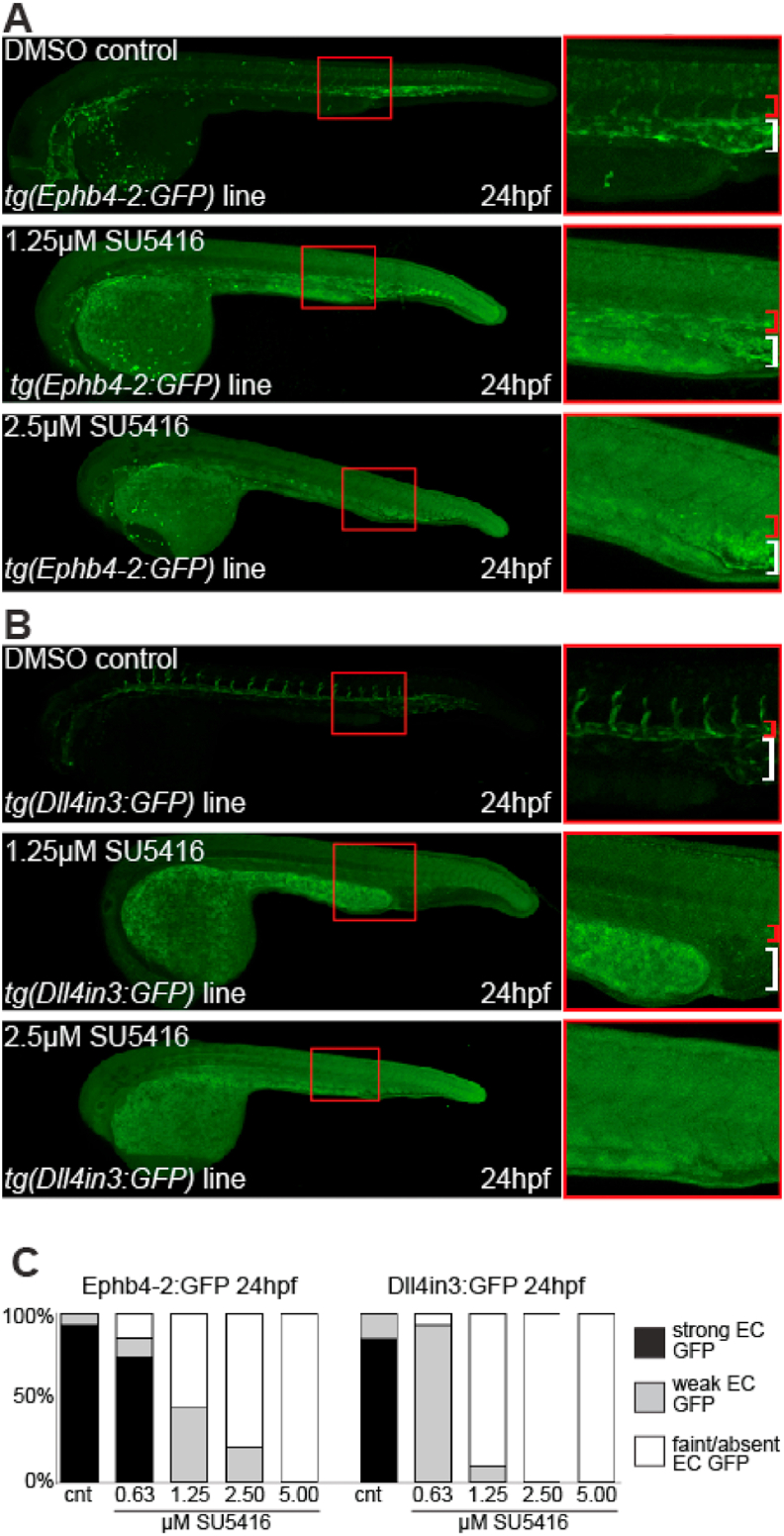
VEGFA signalling is required for both venous and arterial enhancer activity. A-B. Representative 24 hpf venous tg(Ephb4-2:GFP) (A) and arterial/angiogenic tg(Dll4in3:GFP) (B) zebrafish embryos treated with either DMSO control or different concentrations of VEGFR inhibitor SU5416. Red bracket indicates dorsal aorta, white bracket indicates cardinal vein. C. Graph depicting observed GFP expression levels in transgenic embryos treated with DMSO control or different levels of SU5416. Ephb4-2:GFP cnt n ​= ​26, 0.63 ​μM SU5416 n ​= ​27, 1.25 ​μM SU5416 n ​= ​72, 2.5 ​μM SU5416 n ​= ​62, 5 ​μM SU5416 n ​= ​72. Dll4in3:GFP cnt n ​= ​32, 0.63 ​μM SU5416 n ​= ​29, 1.25 ​μM SU5416 n ​= ​31, 2.5 ​μM SU5416 n ​= ​26, 5 ​μM SU5416 n ​= ​33.

ETS factor binding at the Ephb4-2 and CoupTFII-965 venous enhancers is seen to increase after VEGFA stimulation. We therefore next investigated the consequences of inhibiting VEGFA signalling on the activity of venous enhancers in zebrafish. Previous research has suggested that while VEGFA inhibition with higher amounts of SU5416 (e.g 10–20 ​μM) results in EC apoptosis, lower levels of VEGF inhibition result in reduced arterial and venous marker gene expression, although the reduction in venous genes was less pronounced and sometimes compensated by expansion of vein gene activity into the dorsal aorta (Casie Chetty et al., 2017). We therefore determined the consequences of different doses of SU5416 to the venous *tg(Ephb4-2:GFP)* and *tg(CoupTFII-965:GFP)* zebrafish lines compared to the arterial *(tg(Dll4in3:GFP)* zebrafish line. At the lowest concentration of inhibitor, Dll4in3:GFP activity was more notably reduced than either venous enhancer, while higher SU5416 doses significantly reduced activity of all enhancers (Fig. 5 and Fig. S11).

Although Dll4in3:GFP was more sensitive to inhibition of VEGFA signalling than either the venous Ephb4-2:GFP or the CoupTFII-965:GFP, our previous results show that changes in ETS factor binding after VEGFA stimulation can be seen at both venous and arterial enhancers. Consequently, it is unlikely that changes in ETS factor occupancy at enhancers can explain the differences between venous and arterial enhancer responses to VEGFA inhibition. An alternative explanation may be that additional transcription factors (either specifically binding and activating arterial enhancers, or binding and repressing venous enhancers) may be instead responsible for allowing arterial and angiogenic enhancers a greater sensitivity to VEGFA signalling.

### 2.6 VEGFA stimulation is not sufficient to activate the Ephb4-2 and CoupTFII-965 vein EC-specific enhancers

**Fig. 6:**
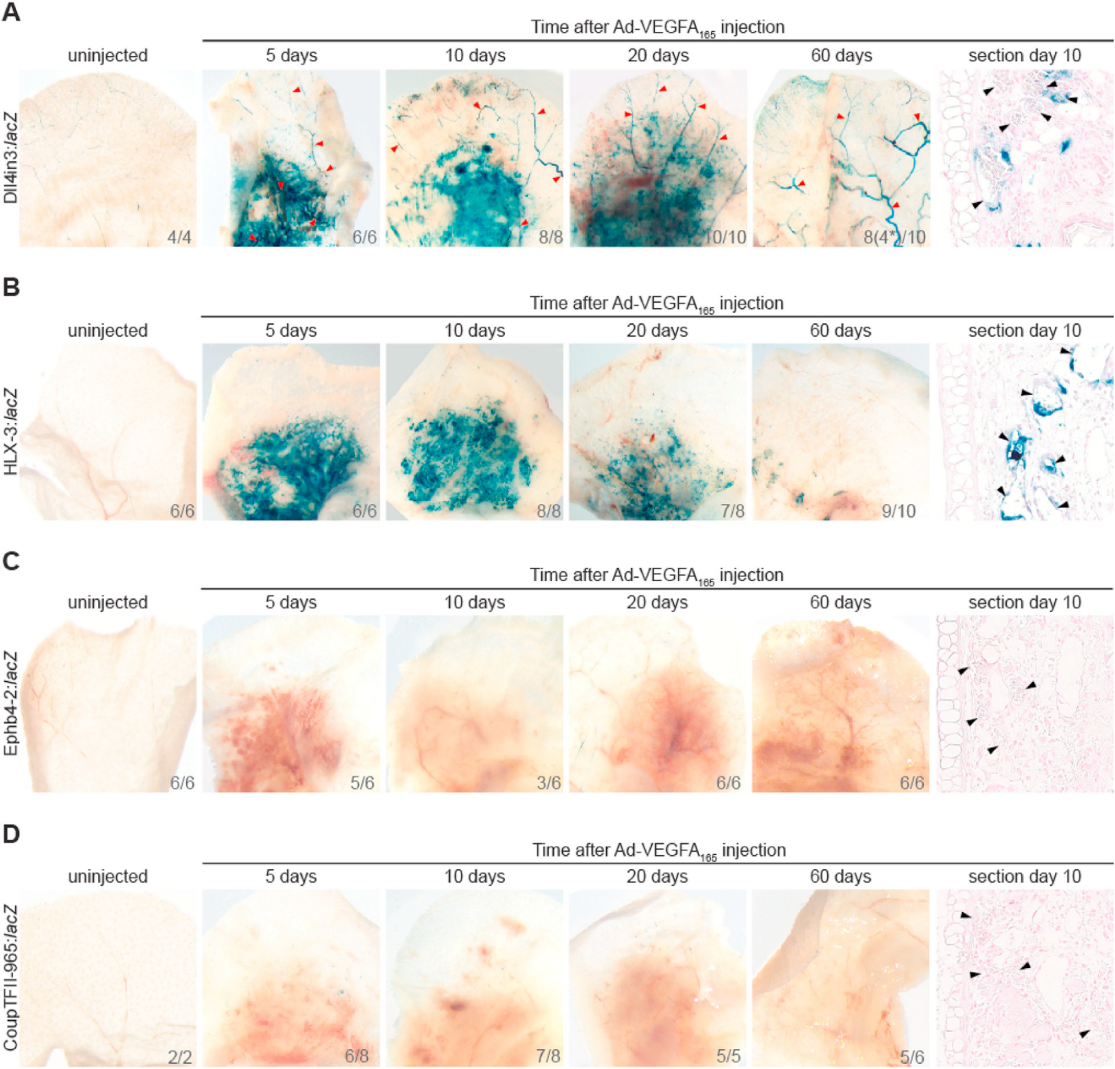
Intradermal injection of Ad-VEGFA164 results in sustained arterial and angiogenic enhancer activity, but venous enhancers were not reactivated. Ad-VEGFA164 was injected intradermally into the ears of adult Foxn1−/− mice transgenic for arterial and angiogenic-expressed Dll4in3:*lacZ* (A), angiogenic expressed HLX-3:*lacZ* (B), and venous-expressed Ephb4-2:lacZ (C) and CoupTFII-965:*lacZ* (D). Enhancer activity was assessed at the stated days after injection by X-gal staining and compared with uninjected control. Red arrowhead ​= ​artery, black arrowhead ​= ​blood vessel. N numbers are indicated on images in bottom right corner, represented as number of ears similar to image shown/total number of ears investigated. Examples of the alternative expression patterns can be seen in Fig. S11.

We next examined whether VEGFA over-expression, and subsequent increased ERG occupancy, is alone sufficient to initiate activity of venous-, arterial- and angiogenic-specific enhancers *in vivo*. We first used an established model of VEGFA-stimulated blood vessel growth in mice, in which an adenovirus expressing VEGFA_164_ (Ad-VEGFA_164_) is injected intradermally into the ears of adult mice (Nagy et al., 2008). This results in robust angiogenesis and vascular differentiation that proceeds in a stereotypical fashion over 60 days (Nagy et al., 2008). Vessel growth in the first 40 days is sensitive to VEGFR inhibition, while vessels at later timepoints are not affected by VEGFR inhibition (Sitohy et al., 2011). To determine if VEGFA was able to equally activate different types of ETS-dependent EC enhancers, we performed intradermal injections of Ad-VEGFA_164_ into the ears of Foxn1^−/−^ nude mice expressing the Dll4in3:*lacZ*, HLX-3:*lacZ*, Ephb4-2:*lacZ* and CoupTFII-965:*lacZ* transgenes. Ad-VEGFA_164_ injections resulted in robust re-activation of the arterial and angiogenic Dll4in3:*lacZ* transgene (Fig. 6A and Fig. S12A-B). Expression of Dll4in3:*lacZ* was seen in arterial structures as well as in punctate ECs throughout the injected areas, assumed to be angiogenic ECs (Fig. 6A). Ad-VEGFA_164_ injection also robustly activated the angiogenic-specific HLX-3:*lacZ* transgene, which was silent in uninjected adult ears. After Ad-VEGFA_164_ injection, the HLX-3:*lacZ* transgene was active in punctate ECs through the injected regions, but was not seen in arterial structures (Fig. 6B and S12C). In both enhancers, angiogenic expression was lost by 60 days after injection, a time-point known to be independent of VEGFA signalling.

These results indicate that Ad-VEGFA_164_ injection can specifically re-activate the Dll4in3 and HLX-3 enhancers in their native EC sub-types. However, they cannot determine whether this occurs via a VEGFA-mediated increase in ERG binding, VEGFA-mediated increase in other transcription factors binding to these enhancers or VEGFA-mediated removal of repressive factor binding. If VEGFA-mediated activation of these enhancers occurs primarily via changes to ETS factors, we would expect a similar reactivation of venous enhancers. We therefore next determined if Ad-VEGFA_164_ injection was able to directly activate the Ephb4-2:*lacZ* and CoupTFII-965:*lacZ* transgenes. Unlike with the arterial and angiogenic enhancers, we observed no endothelial transgene activity at any time point in either Ephb4-2:*lacZ* or the CoupTFII-965:*lacZ* Ad-VEGFA_164_ injected ears, although occasional ectopic expression could be detected (Fig. 6C–D and Fig. S12D-E).

**Fig. 7:**
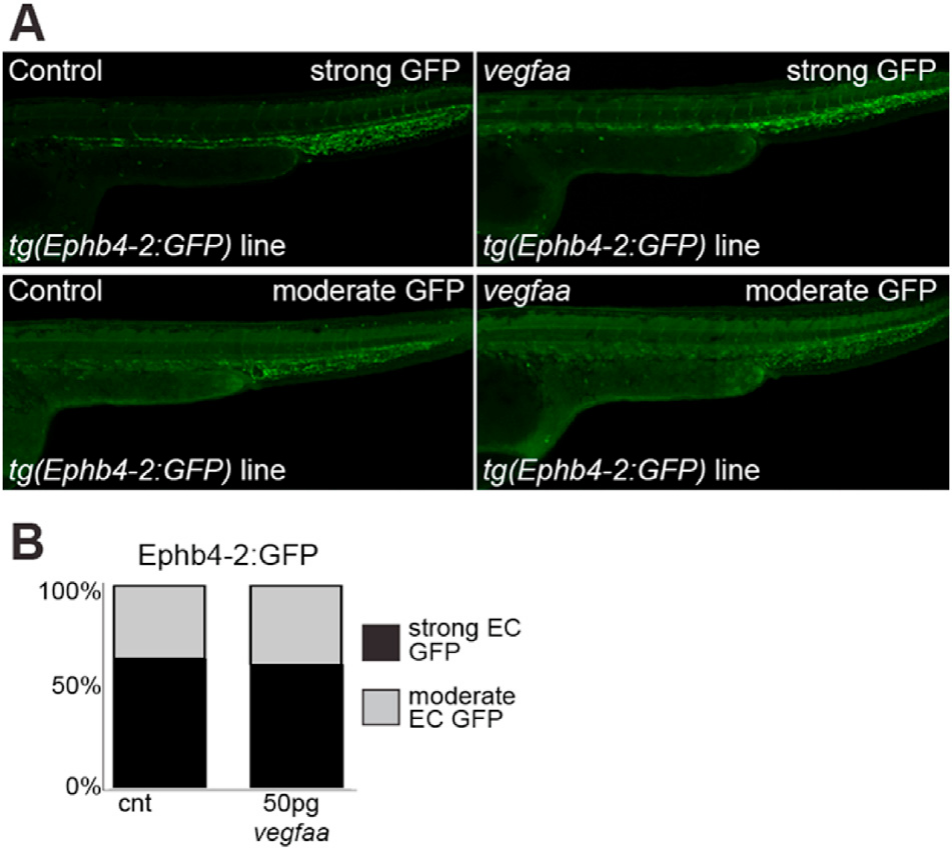
VEGFA overexpression does not change venous Ephb4-2 enhancer activity during embryonic development in zebrafish. A. Representative 28 hpf control (left panel) or *vegfaa* mRNA injected (right panel) *tg(Ephb4-2:GFP)* transgenic embryos show similar levels of expression. B. Graph depicting observed expression pattern of GFP in *tg(Ephb4-2:GFP)* control and *vegfaa* mRNA injected transgenic embryos. Black denotes strong venous GFP expression, grey denotes moderate venous GFP expression. Some variability is seen between embryos in both control and injected groups, but the percentage with strong (upper) and moderate (lower) GFP expression remained similar between the two groups. Control n ​= ​164, injected n ​= ​129. More representative embryos can be seen in Figure S13.

Although developmental arterial and angiogenic enhancers were reactivated in the adult mouse ear by Ad-VEGFA_164_ (Fig. 6A and B), it remains possible that the failure of VEGFA stimulation to ectopically activate venous enhancer:*lacZ* transgenes reflects the absence of a developmental context. Previous work in zebrafish has suggested that over-expression of *vegfaa* can cause arterial gene expression to become more intense, and to expand to the venous compartment, while endogenous venous gene expression was generally reduced (Casie Chetty et al., 2017). Further, over-expression of *vegfaa* in transgenic zebrafish expressing the arterial/angiogenic Dll4enhancer:GFP transgene is reported to induce both increased GFP intensity and expansion of GFP expression into the caudal vein plexus (Wythe et al., 2013). To determine if over-expression of VEGFA can alter Ephb4-2 enhancer activity within the endothelium during development, we injected 50 ​pg *vegfaa*_121_ and *vegfaa*_165_ mRNA into 1-cell stage *tg(Ephb4-2:GFP)* embryos (following protocol and concentration from Casie Chetty et al., 2017) and examined GFP expression at 28 hpf. In agreement with our adult mice data, we observed little difference between the control and injected *tg(Ephb4-2:GFP)* embryos (Fig. 7A–B and S13). This did not change significantly when we increased the amount of vegfaa injected (Fig. S13). This result therefore further indicates that increased VEGFA signalling does not increase Ephb4-2 enhancer activity. Additionally, at 50 ​pg *vegfaa*_121_ and *vegfaa*_165_ we saw no clear expansion of Ephb4-2:GFP expression beyond the venous endothelial expression pattern observed in control embryos (Fig. 7A and B and Fig. S13). In comparison, injecting 50 ​pg *vegfaa*_121_ and *vegfaa*_165_ mRNA into 1-cell stage *tg(Dll4in3:GFP)* embryos resulted in slightly increased GFP intensity and expansion into the caudal vein plexus, as previously described by Wythe et al. (2013) (Fig. S14). The increase in GFP intensity was more notable at higher *vegfaa* levels (Fig. S14). Taken together, these results suggest that the failure of Ad-VEGFA_164_ to activate venous gene enhancers in the mouse ear is unlikely to be a result simply of developmental context. Furthermore, the Ephb4-2:*lacZ* transgenes can be reactivated in injured neonatal hearts (Payne et al., 2019), suggesting that absence of normal activity does not affect enhancer reactivation. Taken together, these results indicate that VEGFA stimulation is not sufficient to activate transcription from the Ephb4-2 venous EC-specific enhancer, despite its reliance on VEGFA-augmented ETS transcription factors.

## 3 Discussion

The role of ETS transcription factors in the regulatory hierarchy of endothelial cells has been unclear. The specification and maintenance of the endothelial cell lineage requires the shared activation and repression of many lineage-defining genes. However, differential gene expression within specific sub-populations of endothelial cells is also essential for vascular function. Consequently, spatial and temporal control of endothelial gene expression must involve multiple layers of regulation. While analysis of arterial and angiogenic-specific enhancers has supported a proposal that ETS factors play a specific role in the activation of arterial and angiogenic genes downstream of VEGFA signalling, ETS factors have also been implicated in the more general activation of genes, and their cognate regulatory elements, involved in endothelial identity and maintenance. However, the analysis of the precise roles played by ETS factors in the vasculature has been complicated by the abundance of different ETS factors in the endothelium coupled with extensive redundancy between different ETS family members. Parsing their two potential functions is further challenged by the multiple roles played by VEGFA signalling in the vasculature. Recent analysis in zebrafish concluded that low levels of VEGFA signalling promotes general endothelial identity and survival, while higher levels of VEGF signalling primarily promotes arterial specification (Casie Chetty et al., 2017). Consequently, while the ability of VEGFA signalling to modify and activate ETS factors has been specifically linked to arterial and angiogenic gene activation, it could equally relate to a more general role for ETS factors in endothelial identity and maintenance.

In this paper, we have clearly demonstrated that vein-specific gene enhancers can be reliant on ETS factors for activation in venous ECs. This is similar to that previously reported in arterial-specific and angiogenic-specific enhancers, even though expression of venous genes is not induced to the same extent by high VEGFA signalling. Further, we have shown that VEGFA signalling can also increases ETS factor binding at venous enhancers, indicating that selective arterial and angiogenic gene activation is unlikely to be achieved through this mechanism. Because our analysis was predominantly restricted to two venous enhancers, the conclusions may not equally apply to all venous-expressed genes. Of note, Casie Chetty et al. (2017) found that perturbation of VEGFA signalling in zebrafish had differing effects on different venous genes, in part due to variable expression boundaries and stringencies (venous-enriched genes with some arterial expression behaved differently to those with more vein-restricted expression patterns). However, this caveat can be equally applied to previous research on the regulation of arterial and angiogenic-specific patterns of gene expression. Additionally, because the enhancers studied here were both vein-specific and ETS-dependent, our analysis is sufficient to conclude that ETS factors are unlikely to alone specifically and selectively activate arterial and angiogenic specific gene expression patterns. These results instead strongly support a model in which VEGFA signalling-induced ETS factor binding contributes to overall endothelial differentiation and identity. Further, this concept of ETS transcription factors as required components of all endothelial gene expression is also supported by recent research showing the ETS factor ERG binds nearly all EC super-enhancers, a class of enhancer that commonly controls the expression of cell identity genes (Kalna et al., 2019). It also agrees with the known role of ETS in combination with Forkhead factors in the regulation of pan-endothelial gene expression during early endothelial differentiation (De Val et al., 2008).

If ETS factors are primarily regulators of general endothelial lineage specification and maintenance, then the spatio-temporal information needed to direct specific patterns of gene expression within different EC subtypes would most likely be provided by other transcription factors either through activation or repression. An essential role for non-ETS factors is supported by a number of studies that have shown the ETS factor motifs, although required for subtype-specific enhancer activation, are not themselves sufficient for their activity: arterial activity of the Dll4in3, ECE1intron, Flk1in10 and NOTCH1+16 enhancers can be entirely ablated by mutations to RBPJ and/or SOXF binding motifs even when ETS motifs within the enhancers are untouched and functional (Chiang et al., 2017; Becker et al., 2016; Robinson et al., 2014; Sacilotto et al., 2013). Likewise, activity of the angiogenic HLX-3 enhancer is ablated by mutations specific to MEF2 motifs, as is angiogenic activity of the Dll4in3 enhancer (Sacilotto et al., 2016). Functional evidence also supports a role for these transcription factors in subtype-specific gene expression: RBPJ, the nuclear effector of Notch signalling, has an established role in both activation of arterial genes and repression of venous genes (Becker et al., 2016; Lawson et al., 2002; Lawson et al., 2001), whilst the SOXF factor SOX17 is required for arterial differentiation in mice and can directly activate *Notch1* expression in arterial ECs (Chiang et al., 2017; Corada et al., 2013). Additionally, knockdown of MEF2 factors in mice is associated with reduced angiogenesis (Sacilotto et al., 2016).

A requirement for SOXF, RBPJ and/or MEF2 factors for arterial and angiogenic gene activity may also explain the ability of VEGFA overexpression to selectively induce Dll4in3 and HLX-3 activity whilst not activating venous enhancers. No identified venous enhancers contain binding motifs for SOXF, RBPJ or MEF2 factors, nor are they known to bind other pan-endothelial enhancers (Sacilotto et al., 2016; Francois et al., 2010; Neal et al., 2019; De Val and Black, 2009). There is also considerable evidence implicating VEGFA signalling upstream of both SOXF/RBPJ and MEF2 factors in the vasculature (Lawson et al., 2002; Sacilotto et al., 2013, 2016). Therefore, whilst VEGFA signalling increases ETS binding at all endothelial enhancers, VEGFA activation of SOXF, RBPJ or MEF2 factors would selectively influence arterial and angiogenic enhancers.

The lack of venous enhancer activation by VEGFA may equally be influenced by the absence of venous induction signals. Similar to arterial and angiogenic enhancers, ETS motifs are not sufficient for the activity of the vein-specific Ephb4-2 and CoupTFII-965 enhancers, as mutations to the SMAD motifs within these enhancers can also entirely ablate all endothelial activity (Neal et al., 2019). Unlike RBPJ and MEF2, SMAD1/5:SMAD4 factors are not downstream of VEGFA signalling, instead requiring phosphorylation downstream of BMP signalling (Hill, 2016).

There is also a potential role for repression in the regulation of both arterial-specific and venous-specific genes. Loss of RBPJ binding results in the expansion of an arterial *Kdr* enhancer into the venous compartment (Becker et al., 2016), and CoupTFII/NR2F2 can play a role in both the activation of venous gene expression and in the repression of arterial genes through recruitment of HDAC1 (Sissaoui et al., 2020). It is therefore also possible that increased ETS binding in response to VEGFA signalling may combine with the loss or gain of a VEGFA-responsive repressive factor binding to help achieve subtype-specific enhancer activity.

Taken together, these results in this paper support a role for VEGFA signalling-induced ETS factor binding in the regulation of endothelial gene expression regardless of their specific expression pattern within the endothelium, whilst differential gene expression within specific sub-populations of endothelial cells is controlled by a combination of additional transcription factors that both selectively activate and repress gene expression.

## 4 Materials and methods

### 4.1 Mice

All animal procedures comply with all relevant ethical regulations, were approved by Clinical Medicine Local Ethical Review Committee, University of Oxford and licensed by the UK Home Office. Stable transgenic mouse lines Tg(Ephb4-2:*lacZ*), Tg(CoupTFII-965:*lacZ*) Tg(Dll4in3:*lacZ*) and Tg(HLX-3:*lacZ*) were generated as previously described (Neal et al., 2019; Sacilotto et al., 2013, 2016). F0 transgenic embryos were generated, dissected and stained in X-gal by Cyagen Biosciences. Yolk sac was collected separately and used for genotyping. For stable transgenic lines, embryos were fixed in 2% paraformaldehyde 0.2% glutaraldehyde and 1X PBS for 60 ​min. After fixation, embryos were rinsed in 0.1% sodium deoxycholate, 0.2% Nonidet P-40, 2 ​mM MgCl_2_ and 1 X PBS, then stained for 2–24 ​h in 1 ​mg/ml 5-bromo-4-chloro-3-indolyo-β-D-galactoside solution (X-gal) containing 5 ​mM potassium ferrocyanide, 5 ​mM ferricyanide, 0.1% sodium deoxycholate, 0.2% Nonidet P-40, 2 ​mM MgCl_2_ and 1 X PBS. After staining, embryos were rinsed through a series of 1 X PBS washes, then fixed overnight in 4% paraformaldehyde at 4 ​°C. All embryos were imaged using a Leica M165C stereo microscope equipped with a ProGres CF Scan camera and CapturePro software (Jenoptik). In instances that images have been altered to improve quality and colour balance, each image within a set have been altered using the same parameters. This occasionally included to selective depletion of the yellow or red colour channel, in order to counteract issues from the X-gal stain solution (which is orange). All samples are stored in 4% PFA indefinitely and slowly become less orange. Consequently, samples stained more recently have a greater yellow/orange hue. An example of this alteration can be seen in (Neal et al., 2019).

### 4.2 Zebrafish

All animal procedures comply with all relevant ethical regulations, were approved by Clinical Medicine Local Ethical Review Committee, University of Oxford and licensed by the UK Home Office. Stable *tg(Ephb4-2:GFP)* and *tg(CoupTFII-965:GFP)* zebrafish lines were generated in (Neal et al., 2019). F0 mosaic transgenic zebrafish embryos were generated using Tol2 mediated integration (Kawakami, 2005). Embryos were maintained in E3 medium (5 ​mM NaCl; 0.17 ​mM KCl; 0.33 ​mM CaCl_2_; 0.33 ​mM MgSO_4_) at 28.5 ​°C. To image, all embryos were dechorionated and anesthetized with 0.01% tricaine mesylate. For analysis of F0 transgenic zebrafish, single embryos were transferred into a flat bottom 96-well plate, and GFP reporter gene expression screened with a Zeiss LSM 710 confocal microscope at 46–50 hpf. Whole zebrafish were imaged using the tile scan command, combined with Z-stack collection under a confocal microscope Zeiss LSM 710 ​MP (Carl Zeiss) at 488 ​nm excitation and 509 ​nm emission (EGFP).

For pharmacological inhibition of VEGF signalling, embryos were manually dechorionated and 0.625 ​μM, 1.25 ​μM, 2.5 ​μM and 5 ​μM of SU5416 (Stratech Scientific Ltd.) added at approximately 5ss as described in (Ferdous et al., 2009). Control embryos were treated with identical concentrations of DMSO without inhibitor. All chemical inhibition experiments were conducted at least three separate times. Analysis was qualitative not quantitative, therefore no statistical analysis was applied to the observations of staining intensity and pattern. Experiments where all zebrafish embryos died were excluded from analysis on assumption of error.

### 4.3 Cloning

Ephb4-2mutETS and CoupTFIImutETS enhancer sequences were initially generated as custom-made, double-stranded linear DNA fragments (GeneArt® Strings™, Life Technologies). DNA fragments were cloned into the pCR8 vector using the pCR8/GW/TOPO TA Cloning Kit (Invitrogen, K2500-20) following manufacturer’s instructions. Once cloning was confirmed, each enhancer was transferred from the pCR8/GW/enhancer entry vector to a suitable destination vector using Gateway LR Clonase II Enzyme mix (Life Technologies, 11791–100) following manufacturer’s instructions. For mouse transgenesis, the enhancer was cloned into the hsp68-LacZ-Gateway vector (provided by N. Ahituv). For zebrafish transgenesis, the enhancer was cloned into the E1b-GFP-Tol2 vector (provided by N. Ahituv).

#### 4.3.1 Ephb4-2 WT

AATCAGTGCGTGCTCGTTAAGTCCTGGAGATCCACTGAGCGCGCAGCCTAACGCTGGAGAAAGTGGTTTGAAACCCAAAGTATAGAAAATGTAAATAAAAGGCAGGCGTGTCAGAGAGGGTGAGGGATCTCCGTAACACCTCATTTCATTTTTTTAAAGGAGGGGGACACTTCCCCGCCGCCTGCAGCCTTGACCTCCAAGGCGGGGGTAGGGACCGTTGTGGCTCTTTCCTGAGGCTGTTTCCTGTCTGGCTCCTGGGGGCCCTCGGGATGGCTGGGAGGGCCCTTCCTCTCATTTGCTAGCACCCCCTCTCATCCATCAGTTTGAGGGGAGGGTCCAGGAAAGACGGCCTCCTATCTACATCAGGGCACTGTGAGTGTGGGGCACGGGATGGTTGGATGAGAGAGGTGCTGTTCCCGAAGTCGGTCCTTTAAGGGCTGCGGTAAGGAGACTTTAATTTAAGGTAATTAGTACAGGGTCTGGAAACTCTGAGGTAGGAGTCTGGGGCACCTGGGAGTCTGCCAAATACCCTAAGGGCGCACACACACACCCCAGCGGGCGACCGGTGATGACCTCTTGTCCGCCTGCGCGCACACACACACCAGCGGGCGCGGGAGACCCGTGATGGCCTTTTGTCCCCGTGCACTTATCTTCCTGGCGCAAGTAGTGCTCCCCACCCCCTGCCCTTCCTCACAGCCCTGCCTGGGTCCCGCTCCGGGGTGGGTCAGCCAGGGCAGGAAACAGCCGGCTTGGCTGGAGCCAGGCTGACCGGCTAGATCTGGGAGTCCCCTCCTCCTTCCCCACGCAGACTCAGGCTCCCCTTCTCTTATCCACAGACACCCCCTTTTTTGCAGCTATCATTCTGCATCCGGGTCCCCCTGAATTTCTGAGTCGTGGCTTGTTCTCAC.

#### 4.3.2 Ephb4-2mutETS

AATCAGTGCGTGCTCGTTAAGTCCTGGAGATCCACTGAGCGCGCAGCCTAACGCTGGAGAAAGTGGTTTGAAACCCAAAGTATAGAAAATGTAAATAAAAGGCAGGCGTGTCAGAGAGGGTGAGGGATCTCCGTAACACCTCATTTCATTTTTTTAAAGGAGGGGGACACTTCCCCGCCGCCTGCAGCCTTGACCTCCAAGGCGGGGGTAGGGACCGTTGTGGCTCTT*ag*CTGAGGCTGTT*ag*CTGTCTGGCTCCTGGGGGCCCTCGGGATGGCTGGGAGGGCCCT*ag*CTCTCATTTGCTAGCACCCCCTCTCATCCATCAGTTTGAGGGGAGGGTCCAGGAAAGACGGCCTCCTATCTACATCAGGGCACTGTGAGTGTGGGGCACGGGATGGTTGGATGAGAGAGGTGCTGTTCCCGAAGTCGGTCCTTTAAGGGCTGCGGTAAGGAGACTTTAATTTAAGGTAATTAGTACAGGGTCTGGAAACTCTGAGGTAGGAGTCTGGGGCACCTGGGAGTCTGCCAAATACCCTAAGGGCGCACACACACACCCCAGCGGGCGACCGGTGATGACCTCTTGTCCGCCTGCGCGCACACACACACCAGCGGGCGCGGGAGACCCGTGATGGCCTTTTGTCCCCGTGCACTTATCT*ag*CTGGCGCAAGTAGTGCTCCCCACCCCCTGCCCT*ag*CTCACAGCCCTGCCTGGGTCCCGCTCCGGGGTGGGTCAGCCAGGGCAGAGAACAGCCGGCTTGGCTGGAGCCAGGCTGACCGGCTAGATCTGGGAGTCCCCTCCTCCTTCCCCACGCAGACTCAGGCTCCCCTTCTCTTATCCACAGACACCCCCTTTTTTGCAGCTATCATTCTGCATCCGGGTCCCCCTGAATTTCTGAGTCGTGGCTTGTTCTCAC.

#### 4.3.3 CoupTFII-965WT

GCTGAGACAAATGGAAAGCTGAAGATAAGGATCCTCTGAGGTGCGAACATACAGCTGTTGGGAATTGCCAGAGAATCGGACCAATAAAGGAAGTCACTATTTTTCCAGGCCTGAAGTGAGTTATAGGGCGAGACGGGTGTTGTATATTTATGTAAGGCAACAGCAGGGAGTTTAAGCGGCTGGATATTGCTGAAAGAGCATCATTCACATTCAGGCGGAGACAAAAGGTGGAAATGAAGCAACATCCTGGCCAAAGAAGGCCTCAAGACAGAATAATAACAGTTCAGAGAGGGGGGCTGTGTGCACGGCCGAGGGTCGGCCTCAAAACCAGGAAATGATCGAGATGCCTTGTCAGATCTTC.

#### 4.3.4 CoupTFII-965mutETS

GCTGAGACAAATGGAAAGCTGAAGATAAGGATCCTCTGAGGTGCGAACATACAGCTGTTGGGAATTGCCAGAGAATCGGACCAATAAAtcAAGTCACTATTTTTCCAGGCCTGAAGTGAGTTATAGGGCGAGACGGGTGTTGTATATTTATGTAAGGCAACAGCAGGGAGTTTAAGCGGCTGGATATTGCTGAAAGAGCATCATTCACATTCAGGCGGAGACAAAAGGTGGAAATGAAGCAACagCTTGGCCAAAGAAGGCCTCAAGACAGAATAATAACAGTTCAGAGAGGGGGGCTGTGTGCACGGCCGAGGGTCGGCCTCAAAACCAtcAAATGATCGAGATGCCTTGTCAGATCTTC.

### 4.4 ClustalW and sequence motif analysis

Mouse and human sequences of putative enhancers were aligned using ClustalW (Thompson et al., 1994). Binding motifs for ERG was obtained from (Wei et al., 2010) and annotated by hand.

### 4.5 Electrophoretic mobility shift assay

Proteins were made using the TNT Quick Coupled Transcription/Translation system as described in the manufacturer’s directions. The truncated ETS1 DNA binding domain (ETS-DBD) and full length ERG were in the pCITE2 plasmid, and transcribed using T7 polymerase. To make each probe, double stranded oligonucleotides with CTAG 5′ overhangs were labelled with 32P-dCTP using Klenow (Promega) to fill in overhanging 5′ ends, and purified on a non-denaturing polyacrylamide-TBE gel. 20 ​μl binding reactions consisted of 2–3 ​μl protein or lysate control, 2 ​μl 10X binding buffer (40 ​mM KCl, 15 ​mM HEPES pH 7.9, 1 ​mM EDTA, 0.5 ​mM DTT, 5% glycerol) and 0.5 ​μg poly dI-dC. For competitor lanes, a 100-fold excess of competitor DNA was added in a volume of 1 ​μl. Binding reactions were incubated at room temperature for 10 ​min before the addition of radiolabelled probe, after which they were incubated an additional 20–30 ​min. Gels were electrophoresed on a 10% non-denaturing polyacrylamide gel. Sequences of the probes and competitor regions are listed below, with *italic underlined* nucleotides modified (GGA to TCA or TCC to TGA) in mutant version:

ETS control probe (De Val et al., 2008) CTAGtaaacccggaagtgtagtacatctggatcg; Ephb4-2 ETS-a CTAGagggggacact*tcc*ccgccg;

Ephb4-2 ETS-b CTAGgttgtggctctt*tcc*tgaggctg.

Ephb4-2 ETS-c CTAGgaggctgtt*tcc*tgtctggc.

Ephb4-2 ETS-d CTAGggccctcg*gga*tggctggga.

Ephb4-2 ETS-e CTAGagggccct*tcc*tctcatttg.

Ephb4-2 ETS-f CTAGgtggggcacg*gga*tggttgg.

Ephb4-2 ETS-g CTAGgatggtt*gga*tgagagaggtgc.

Ephb4-2 ETS-h CTAGcacttatct*tcc*tggcgcaagta.

Ephb4-2 ETS-I CTAGcctgccct*tcc*tcacagccc

Ephb4-2 ETS-j CTAGagccagggca*gga*aacagcc.

Coup-965 ETS-a CTAGacaaat*gga*aagctgaagataa.

Coup-965 ETS-b CTAGgctgaagataa*gga*tcctctgag.

Coup-965 ETS-c CTAGagctgttg*gga*attgccagaga.

Coup-965 ETS-d CTAGcggaccaataaa*gga*agtcactat.

Coup-965 ETS-e CTAGaaggt*gga*aatgaagcaacatc.

Coup-965 ETS-f CTAGaagcaaca*tcc*tggccaaag.

Coup-965 ETS-g CTAGcaaaacca*gga*aatgatcgagatc.

Coup-965 ETS-h CTAGgtcactatttt*tcc*aggcctg.

### 4.6 Chromatin immunoprecipitation (ChIP)

For VEGF stimulation experiments in cells, human umbilical vein pooled endothelial cells (HUVEC, PromoCell, C-12203, between passage 3–6) were grown in Endothelial Cell Growth Medium 2 with BulletKit (PromoCell). Media was changed every 48 ​h. Four 80% confluent 15 ​cm dishes per condition were serum starved in 0.5% Foetal Bovine Serum (Gibco) overnight before being stimulated with VEGFA_165_ (PeproTech) at 25 ​ng/ml for 1.5 ​h. Cells were then trypsinised and the cell pellet collected.

Chromatin immunoprecipitation was carried out as previously described (Neal et al., 2019). Briefly cells were crosslinked for 12 ​min in 0.6% methanol-free formaldehyde (Pierce) room temperature then quenched with glycine to a concentration of 0.2M. Cells were lysed in cell lysis buffer (50 ​mM Tris-HCl (pH8.0), 10 ​mM EDTA, 10 ​mM sodium butyrate, 1% SDS, 0.5 ​mM PMSF and cOmplete, EDTA-free protease inhibitor cocktail (Roche)). Chromatin was sheared by sonication to a mean chromatin fragment size of 650–850bp using a Covaris sonicator (S220). Sonicated chromatin was incubated overnight in ChIP dilution buffer (16.7 ​mM Tris(pH8.0), 167 ​mM NaCl, 1.2 ​mM EDTA, 1% Triton X – 100, 0.01% SDS) with 4 ​μg of ERG antibody (Abcam ab110639) or Rabbit IgG control (Cell Signalling Tecgnoloogy # 3900S) with a no-antibody control. Immunoprecipitation was performed with Dynabeads-protein G (ThermoFischer), and blocked overnight in 0.5 ​mg/ml bovine serum albumin (Sigma-Aldrich). G-Dynabead immunocomplexes were washed three times with low-salt buffer (20 ​mM Tris-HCL (pH8.0) 150 ​mM NaCl, 2 ​mM EDTA, 1% Triton X-100, 0.1% SDS), high-salt buffer (20 ​mM Tris-HCl (pH8.0) 500 ​mM NaCl, 2 ​mM EDTA, 1% Triton X-100, 0.1% SDS) and LiCl buffer (250 ​mM LiCl, 0.5% NP-40, 0.5% sodium deoxycholate, 1 ​mM EDTA, Tris–HCl 10 ​mM, pH 8.0). Beads were eluted in 0.2 ​ml elution buffer and ChIPed-DNA was reverse crosslinked overnight at 55 ​°C in elution buffer plus 0.3M NaCl (final concentration), 20 ​μg RNase A (Invitrogen) and 20 ​μg proteinase K (Fermentas). DNA was column purified with QIAquick PCR purification Kit (Qiagen).

Immunoprecipitated DNA was analyzed by qPCR using TaqMan Custom Gene Expression Assay Probes (ThermoFischer) designed against 100bp regions of the Ephb4-2 enhancer, the CoupTFII-965 enhancer or a gene dessert region of Chromosome 7 as a negative control.

#### 4.6.1 TaqMan_Probe Ephb4-2

ACCCCTGCCCTTCCTTGCTGTTCTGCCTGGGTCCTGCGCCCGGGTTGGGGGGGGTGGGCCGGTCACCGAGGGCAGGAAACAGCCGGCTTCACTGGAGCCAGGCAGACCAG.

#### 4.6.2 TaqMan_Probe CoupTFII-965

AGCGGCTGTATATTGCTGAAAGAGCATCATTCACATTCAGGCAGAGACAAAAGGTGGAAATGAAGTAACATCCTGGCTGAAGAAGGCCTCACGACAGAATA.

#### 4.6.3 TaqMan_Probe negative control

CCTCAGCCTCCCAAGTAGCTGGGATTACAGGTGTGTGCTACCATGCCTGGCTAATTTTTGTATTTTTAGTAGAGACAGGGTTTCACCATGTTGGCCAGGCTGGTCTCGAACTCCTGAACTCAGG TGATCTA.

Each ChIP was performed on at least three biological replicates, with three technical replicates for each. Statistical analysis was performed in StepOne plus software, Microsoft Excel. Input was taken as the supernatant from the non-antibody control condition. Results are expressed as the mean of the % input defined as 100∗(2^(adjusted Input ct – ct IP)) across all replicates. Significant differences were calculated using ANOVA f test with p values subsequently derived from Tukey HSD test, accounting for multiple comparison correction. Graphs were produced using R[] statistical package.

### 4.7 ChIP-seq data analysis

ChIP-seq analysis was conducted on the published and publicly available data from Chen et al. (2017). Data was accessed from the NCBI Gene Expression Omnibus (GEO, https://ncbi.nlm.nih.gov/geo/) under accession GSE93030. The four ChIP-seq datasets used have accession numbers GSM2442775 to GSM2442778. Data consisted of ChIP-seq Model-based Analysis of ChIP-Seq (MACS) (Chen et al., 2017) regions with peak values. These MACS regions are relative to the GH37 human genome. The ChIP-seq results in the enhancer regions of interest (ROI) were extracted using BEDTools v2.29.2 (Quinlan and Hall, 2010). MACS peak heights were then plotted, using R version 4.0.1 and libraries (https://CRAN.R-project.org/package = data.table and https://CRAN.R-project.org/package=ggpubr). GRCh37 ROIs: HLX-3 chr1:221,049,659–221,050,776, Dll4in3 chr15:41,222,807–41,223,778, Ephb4-2 chr7:100,426,194–100,427,393, and CoupTFII-965 chr15:95,908,708–95,909,301.

### 4.8 Morpholinos (MOs)

Antisense MOs were ordered from GebeTools LLC and dissolved in water before injected into 1–2 ​cell stage zebrafish embryos as previously described (Sacilotto et al., 2013). Sequences used were:

*fli1* MO (3 – 6 ​ng) TTTCCGCAATTTTCAGTGGAGCCCG (Liu and Patient, 2008).

erg MO (3 – 6 ​ng) CAGACGCCGTCATCTGCACGCTCAG (Ellett et al., 2009).

### 4.9 In situ hybridization in zebrafish

For zebrafish whole-mount *in situ* hybridization *ephb4*, *efnb2 and stab1l* probes were generated as custom-made, double-stranded linear DNA fragments (GeneArt® Strings™, Life Technologies), cloned into the pCR2 vector using the TOPO/TA cloning kit (Invitrogen 450641) and transcribed using SP6 and T7. The sequences are provided below. *dll4* probe was kindly provided by R. Patient, University of Oxford, Oxford. Whole-mount *in situ* hybridization was conducted as previously described (Neal et al., 2019). Briefly, embryos were collected at 28hpf and fixed overnight at 4 ​°C in 4% PFA. Fixed embryos were dehydrated and stored at −20 ​°C in 100% methanol. Before use, embryos were rehydrated in 1 x PBS with 0.1% Tween-20 (PBST) and made permeable by digestion with 15 ​μg/ml proteinase K (Sigma-Aldrich) for 10 ​min (28hpf embryos) followed by two PBST washes. The embryos were then fixed in 4% PFA for 20 ​min and thereafter washed five times with PBST. Embryos were transferred into hybridization solution (50% formamide, 5 x SSC, 0.1% Tween 20, 50 ​μg/ml heparin, 500 ​μg/ml of tRNA adjusted, 10 ​mM citric acid) for 2 ​h at 65 ​°C, transferred into diluted antisense riboprobe/hybridization solution and incubated overnight at 65 ​°C. Probes were removed and embryos relocated to a Biolane HT1 *in situ* machine (Intavis Bioanalytical Instruments). Embryos were washed through a dilution series of 2 x SSC followed by 0.2 x SSC at 65 ​°C and thereafter taken through room temperature dilution washes of 100% MABT (0.1M Maleic Acid, 0.15M NaCl, pH 7.5). Nonspecific sites were blocked with MAB block (MABT with 2% Boehringer block reagent) and the embryos incubated for 15 ​h with 1:4000 antiDIG antibody (Roche) at 4 ​°C, before washing in MABT. Prior to staining, embryos were washed in AP buffer and the *in situ* signal developed at room temperature with BM Purple (Sigma-Aldrich). Staining was stopped as appropriate, and embryos were bleached in 3% H_2_O_2_/0.5% KOH until pigmentation disappeared, then re-fixed in 4% PFA for 20 ​min and washed 4 times with PBST. Embryos were transferred to 80% glycerol for imaging.

Analysis was qualitative not quantitative, therefore no statistical analysis was applied to the observations of staining intensity and pattern. Numbers of zebrafish embryos was no less than 30/*in situ*/condition. Experimental blinding was not used as phenotypes of control and treated were easily detectable due to morphological defects.

#### 4.9.1 Zebrafish *ephb4a in situ* probe

TCTCAGCTCTGGACAAGCTGATCCGCAACCCGGCCTCACTCAAAATCACAGCGCAGGAGGGGGCGGGCCCCTCTCACCCTCTGCTGGACCAGCGGTCTCCACTCACGCCCTCATCCTGCGGGACAGTGGGTGACTGGCTGCGGGCCATCAAGATGGAGCGCTACGAGGAGACATTTCTGCAGGCGGGATACACGTCCATGCAGCTCGTCACCCACATCAACACGGAGGATCTGCTGCGTTTGGGAATAACTTTAGCAGGTCACCAGAAGAAGATTCTCTCCAGCATTGAGGCTCTCGGGATTCAAAACAAAGCACCAGGGAATGTGCTGTACTGA.

#### 4.9.2 Zebrafish *efnb2a in situ* probe

AAAACCAAGTCGATGAAAATCATCATGAAGGTTGGACAAAACCCCTCTGATCCCATTTCCCCCAAAGACTACCCTACCAGTTACCCTCCCAAACACCCTGACTTAGGGGGCAAGGACAGCAAATCGAATGAAGTACTTAAGCCAGATGCATCTCCTCATGGGGAAGATAAGGGAGATGGAAATAAATCCTCATCAGTCATTGGCTCAGAGGTGGCCCTGTTTGCCTGCATCGCCTCAGCAAGCGTCATCGTCATCATCATAATCATCATGCTAGTTTTCCTTCTCCTGAAGTATCGACGA.

#### 4.9.3 Zebrafish *stab1l in situ* probe

GGATTCAGCAGCTACAGACACACCCAACCTCATCGACTAGCACAGACAGCAGCGTTAAACTCTCCCTTCATTTACCTGCAATCAGCTGACCGCTCTTAAAATAAAGGTTCTGTATTGGCATTGATGGTTCCGCGAAGAATCTTTATAAGCCATAACATCTTTCCATTTCCATGAGGTGTAAAAAGACTCTTTAGAATATTAAAATGTTACTTCATAAACATTTGATGTGTTTGATTGCAGATACTTCAGAGTGTTTAACTTCCACCCATTTATTTCTGCGTTTCACACATATTTTTGACTAAAAATGTTCTTTACATTAAGAAAAAATGGTGTACTCACCCTCAAGTAGGTCCAAACCTTCACGAGTTTCTTTTTTTGCCTTCTGTTGAACACAAACAAGAAAATATTTTGATAATGCGTTAAGCAAGGGGCCATTAGCTGTTTTGATCCAACTTTTTTCATGCGCATTTTAAATTATCGCATGTAAAAAAGCTTAATGGAAACCCAAGATGTGCTTAATTTGTCAAAATCTGCAT.

### 4.10 Ad-VEGFA_164_ intradermal ear injections

Intradermal Ad-VEGFA_164_ injections were performed on nude mice as described in (Nagy et al., 2008). Briefly, mice were injected on the dorsal side of the ear with 10 ​μl of Ad-VEGFA_164_ (provided by Lilly) diluted 1:30 in sterile 3% glycerol/PBS. At the required time-point after injection, ears were harvested and skin removed from the dorsal side. Ears were fixed in 2% paraformaldehyde and 0.2% glutaraldehyde in PBS for 20 ​min at 4 ​°C, washed twice in PBS then X-gal stained overnight at room temperature. Ears were then placed in 4% paraformaldehyde for storage.

For histological analysis of Ad-VEGFA_164_ injected ears, ears were harvested and X-gal stained as described above, then dehydrated through a series of ethanol washes, cleared by xylene and paraffin wax-embedded. 5 or 6-μm sections were prepared and de-waxed. For imaging of X-gal staining, slides were counterstained with nuclear fast red (Electron Microscopy Sciences).

### 4.11 VEGF overexpression

pCS2+*vegfaa*_*121*_ and pCS2+*vegfaa*_*165*_ plasmids were kindly provided by S. Sumanas, Cincinnati Children’s Hospital Medical Center, Ohio, USA. *vegfaa*_*121*_ and *vegfaa*_*165*_ mRNA was synthesised *in vitro* using the mMessage mMachine SP6 transcription kit (ThermoFisher Scientific) and injected into 1 ​cell stage zebrafish embryos at a final concentration of 50 ​pg of mRNA per embryo. Analysis was qualitative not quantitative, therefore no statistical analysis was applied to the observations of staining intensity and pattern.

## Declaration of competing interest

No competing interests declared.

## Data Availability

The authors declare that the main data supporting the findings of this study are available within the article, its Supplementary Figures and Methods.

## Research articles

the total length of the article should not exceed 7000 words, including the main text and figure legends, but not the title page, abstract, materials and methods section or reference list (reference citations in the text do count towards the word limit).
