# Supplementary material for: ETS factors are required but not sufficient for specific patterns of enhancer activity in different endothelial subtypes
Source: Dev Biol. 2021 May;473:1–14. doi: 10.1016/j.ydbio.2021.01.002 (PMC8026812; doi:10.1016/j.ydbio.2021.01.002)
Supplement: Multimedia component 1 [file mmc1.docx]

**
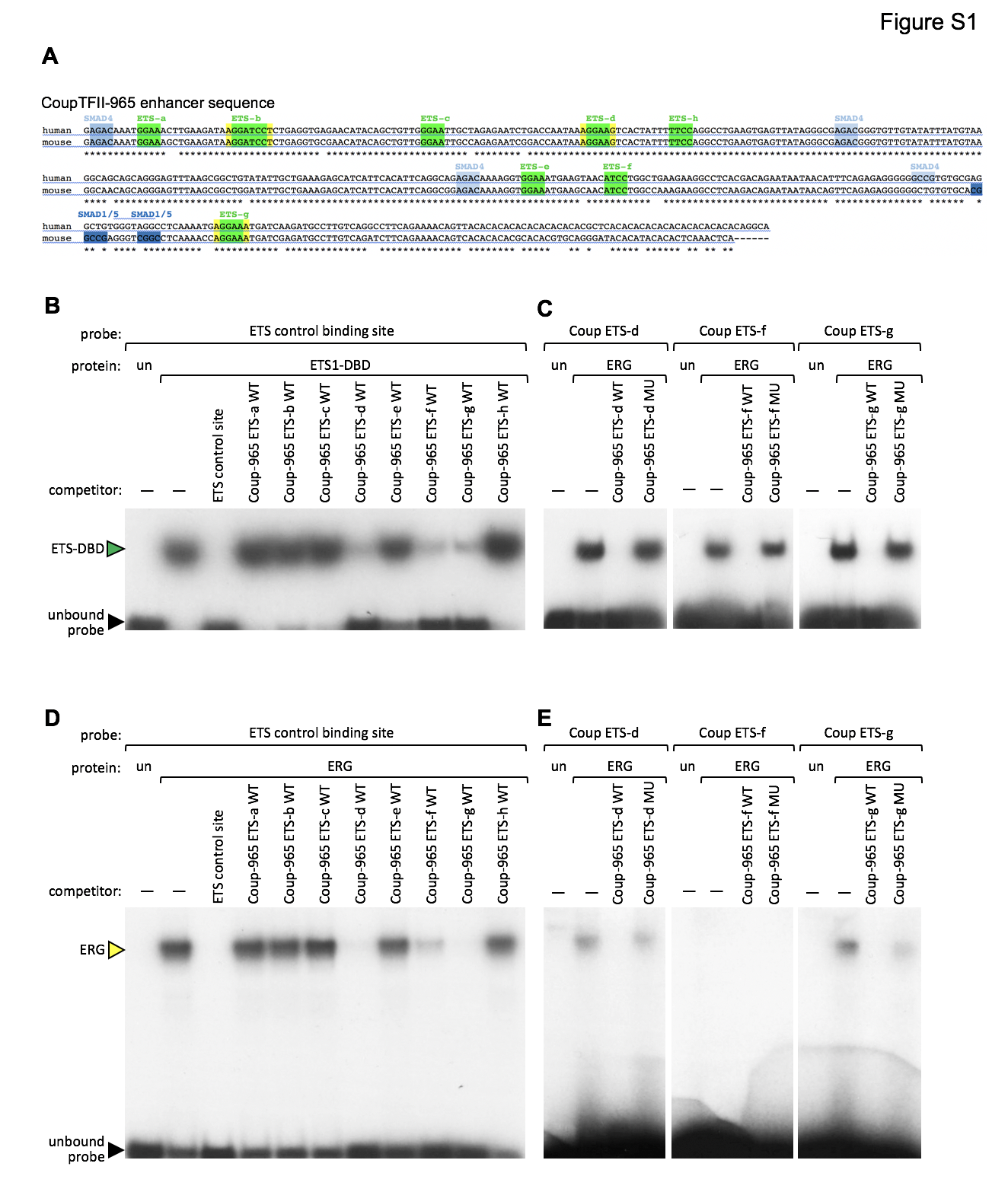
**

**Figure S1,** relating to Figure 1 in main text

**The CoupTFII-965 venous enhancer contains functional ETS motifs**

**A.** ClustalW alignment of the human and mouse sequences of the CoupTFII-965 enhancer annotated with conserved ETS binding motifs (green), SMAD4 binding motifs (light blue) and SMAD1/5 binding motifs (dark blue) as previously reported (Neal et al., 2019). Flanking regions outside core ETS binding motifs which adhere to the ERG consensus motifs are indicated in yellow. * denotes nt conserved between human and mouse sequences.

**B-E.** Radiolabelled oligonucleotide probe encompassing a known ETS binding motif (ETS control consensus binding site, **B** and **D**) or putative CoupTFII-965 ETS motif (ETS-d, ETS-f and ETS-g, **C** and **E**) was incubated with either unprogrammed TNT lysate (un), recombinant ETS1 DNA binding domain protein (ETS1-DBD, **B-C**) or ERG protein (**D-E**). Competitors added were either water control (-), an excess of unlabelled self-probe (ETS control site) or a single putative CoupTFII-965 ETS wildtype (WT) or mutant (MU) motif. Gel shifts denoting protein binding are indicated by green (ETS-DBD) and yellow (ERG) arrowheads, unlabelled probe is indicated by black arrowhead.

**
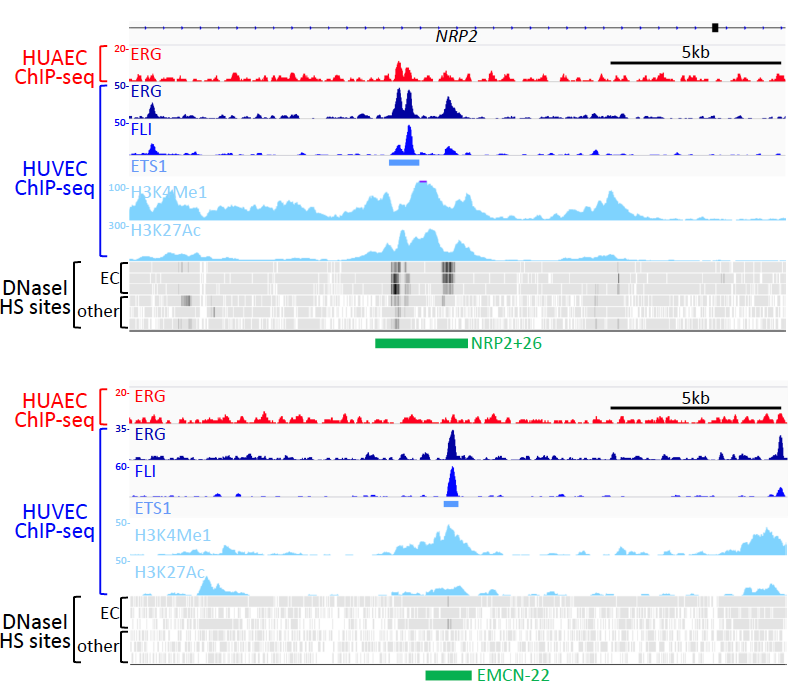

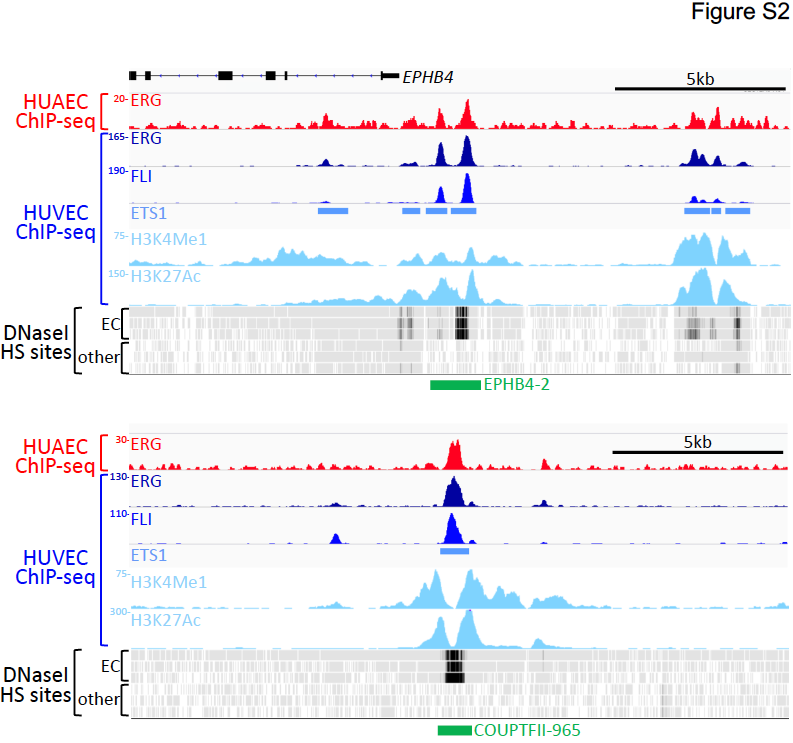
**

**
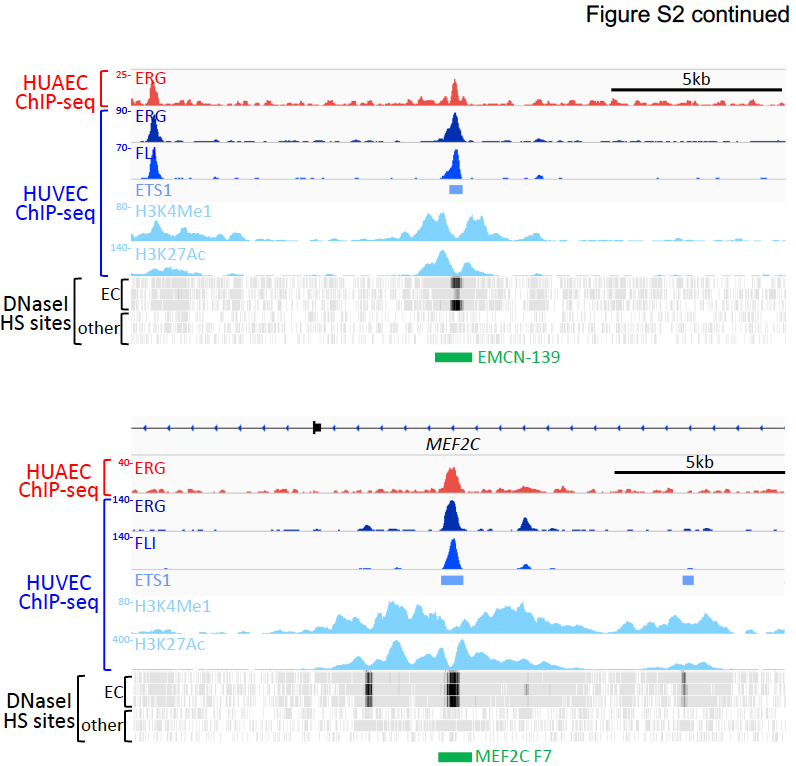
**

**Figure S2**, relating to Figure 1 in main text.

**ETS factor binding, enhancer-associated histone modifications and DNase I hypersensitivity at venous enhancers**

IGV genome browser view of ERG binding from (Sissaoui et al., 2020) (HUAEC in red, HUVEC in dark blue), FLI binding from (Nagai et al., 2018) (in HUVECs, royal blue) and ETS1 binding from (Chen et al., 2017) (in HUVEC after 4 hours VEGFA, lighter blue horizontal line) aligned to UCSC genome browser view of enhancer histone marks (pale blue, in HUVECs), and DNase I HS (black heat map, HUVECs and HMVECs). The human orthologues of Ephb4-2, CoupTFII, Nrp2+26 (all from (Neal et al., 2019)), Mef2CF7 (venous specificity described in (Zhou et al., 2017)) and the EMCN-22(Neal et al., 2019) and EMCN-139 (Kanki et al., 2011) venous enhancers are indicated in green horizontal lines.


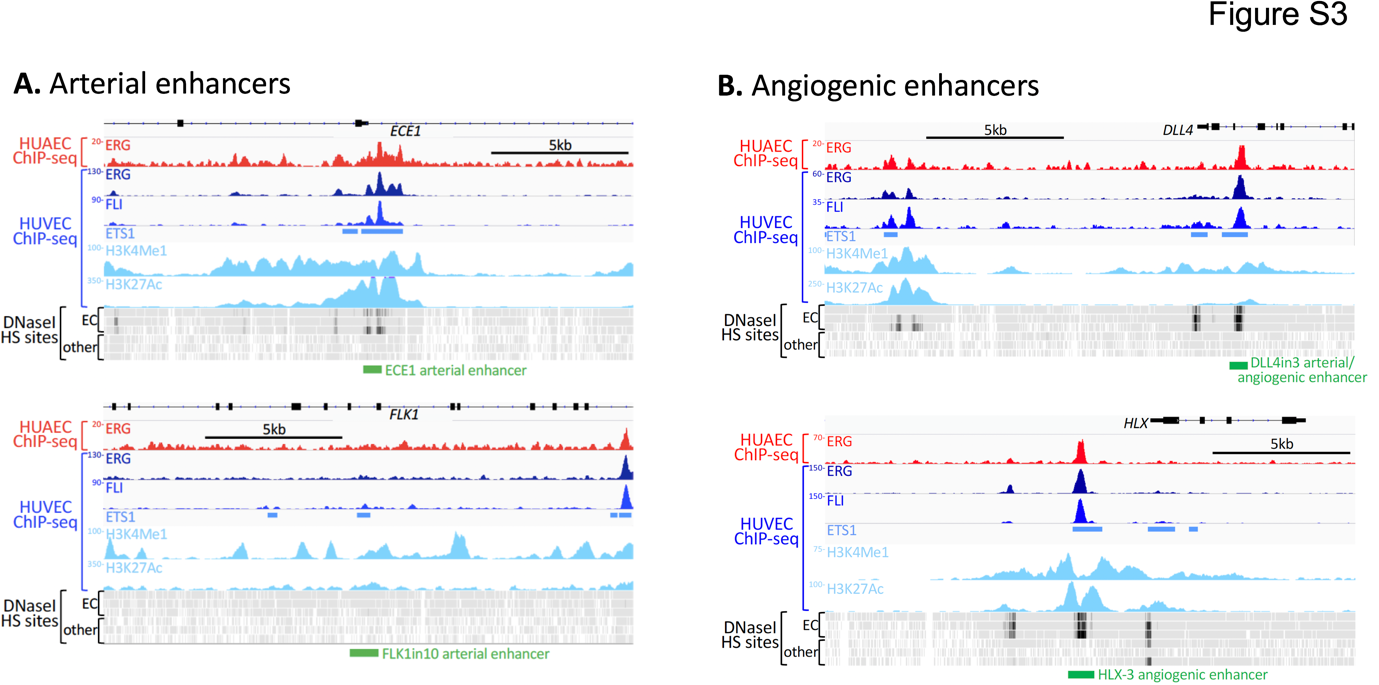


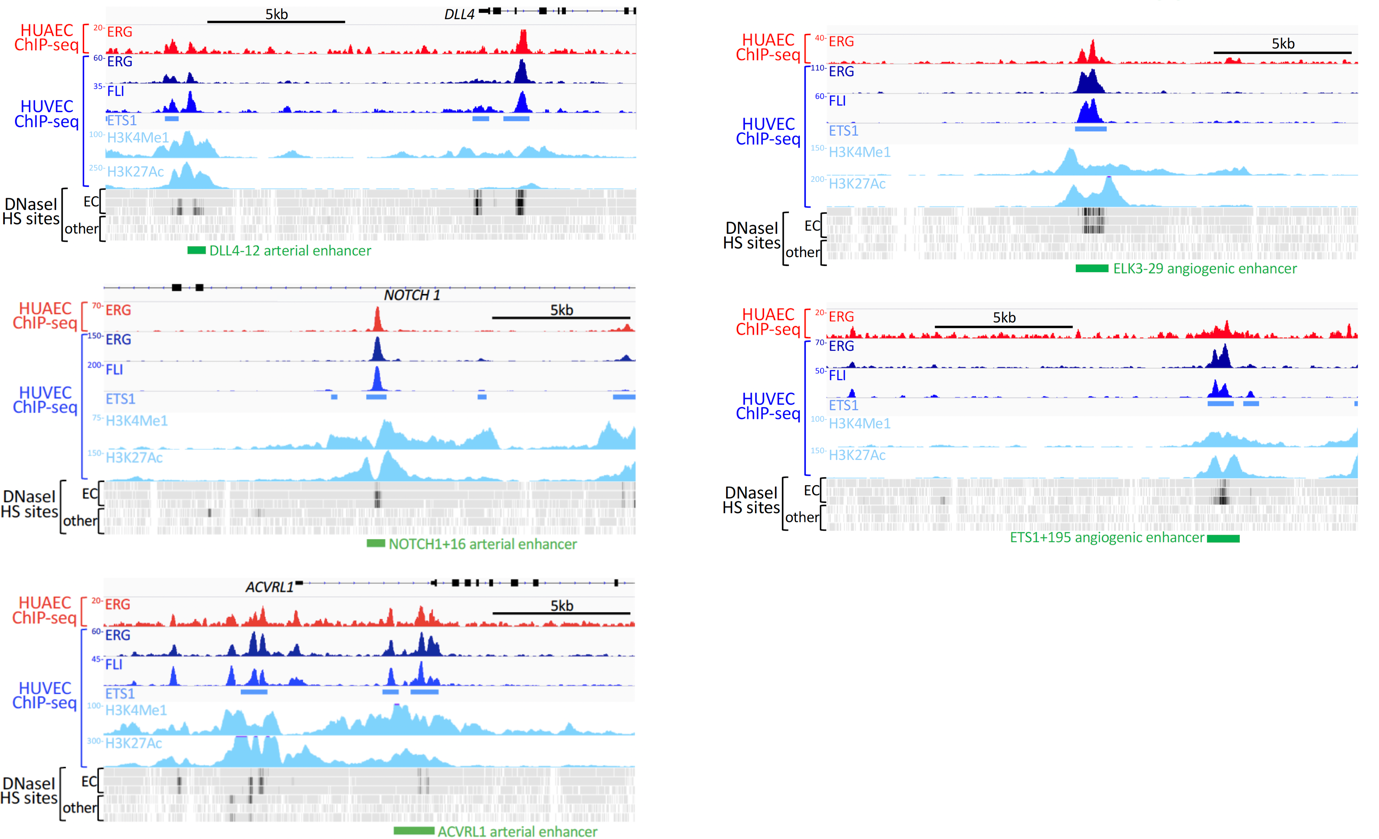


**Figure S3**, relating to Figure 1 in main text.

**ETS factor binding, enhancer-associated histone modifications and DNase I hypersensitivity at arterial-specific and angiogenic-specific enhancers**

**A-B**. IGV genome browser view of ERG binding from (Sissaoui et al., 2020)(HUAEC in red, HUVEC in dark blue), FLI binding from (Nagai et al., 2018) (in HUVECs, royal blue) and ETS1 binding from (Chen et al., 2017) (in HUVEC after 4 hours VEGFA, lighter blue horizontal line) aligned to UCSC genome browser view of enhancer histone marks (pale blue, in HUVECs), and DNase I HS (black heat map, HUVECs and HMVECs). **A** The human orthologues of Ece1 (Robinson et al., 2014), Flk1in10 (Becker et al., 2016), Dll4-12 (Sacilotto et al., 2013) arterial enhancers, and the NOTCH1+16

(Chiang et al., 2017) and ACVRL1 (Seki et al., 2003) arterial enhancers are indicated by green horizontal lines. **B** The human orthologue of Dll4in3 arterial and angiogenic enhancer (Sacilotto et al., 2013), and the HLX-3, ELK3-29 and ETS1+195 (Sacilotto et al., 2016) angiogenic enhancers are indicated by green horizontal lines.


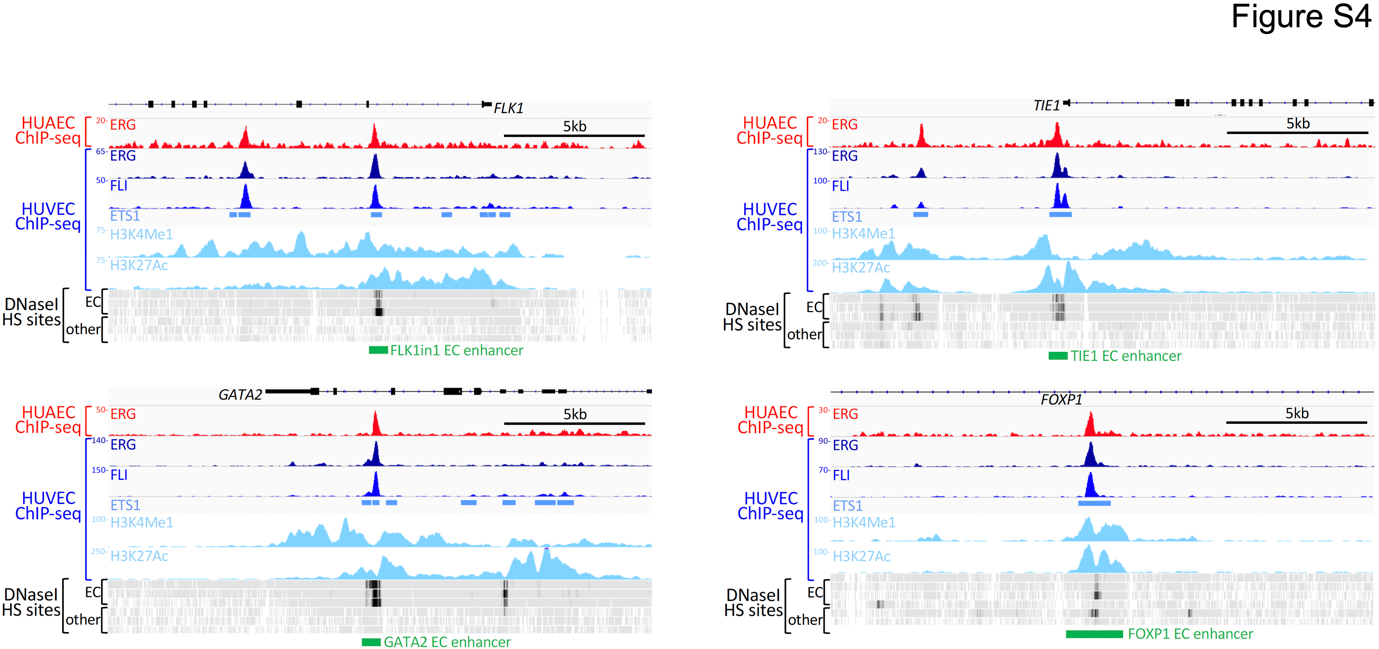

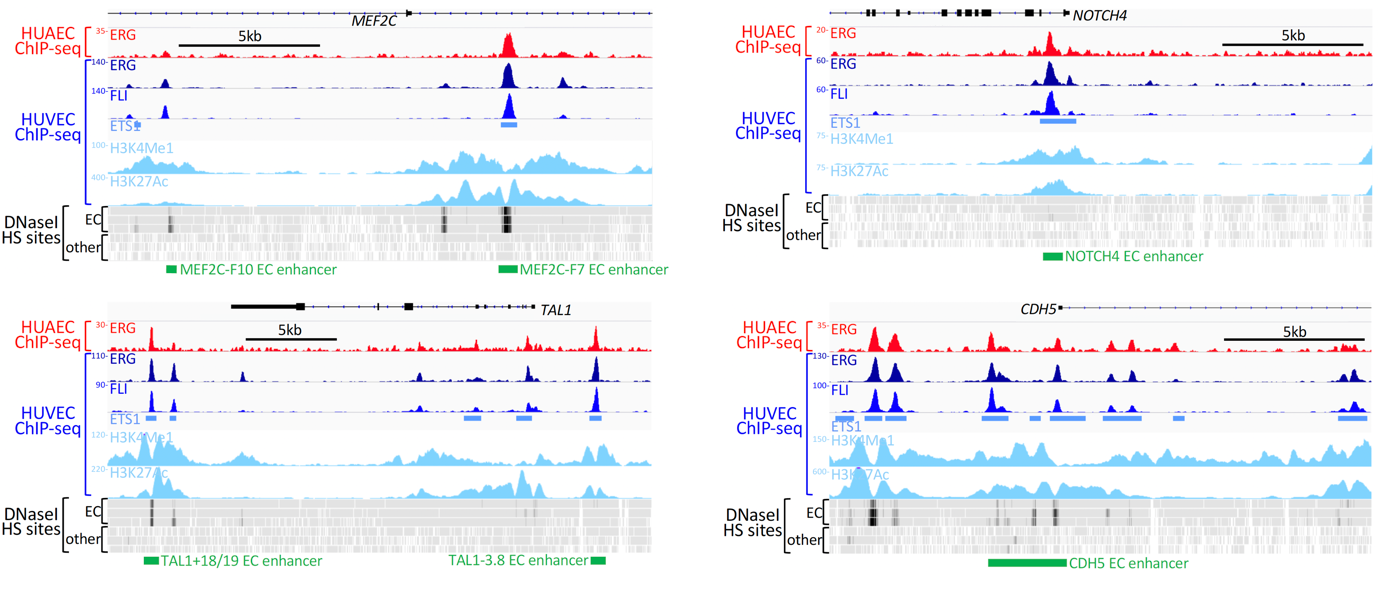


**Figure S4**, relating to Figure 1 in main text.

**ETS factor binding, enhancer-associated histone modifications and DNase I hypersensitivity at enhancers activate in all endothelial cells**

IGV genome browser view of ERG binding from (Sissaoui et al., 2020) (HUAEC in red, HUVEC in dark blue), FLI binding from (Nagai et al., 2018) (in HUVECs, royal blue) and ETS1 binding from (Chen et al., 2017) (in HUVEC after 4 hours VEGFA, lighter blue horizontal line) aligned to UCSC genome browser view of enhancer histone marks (pale blue, in HUVECs), and DNase I HS (black heat map, HUVECs and HMVECs). The human orthologues of Flk1 (Kappel et al., 1999), Gata2 (Khandekar et al., 2007), Mef2C-F10 (De Val et al., 2008), Tal1+18/19 (Sánchez et al., 1999), Tal1-3.8 (Göttgens et al., 2004) and Tie1 (Boutet et al., 2001) pan-endothelial enhancers, and the FOXP1 (De Val et al., 2008), NOTCH4 (Wu et al., 2005) and CHD5 (Prandini et al., 2005) pan-endothelial enhancers are indicated by green horizontal lines.

**
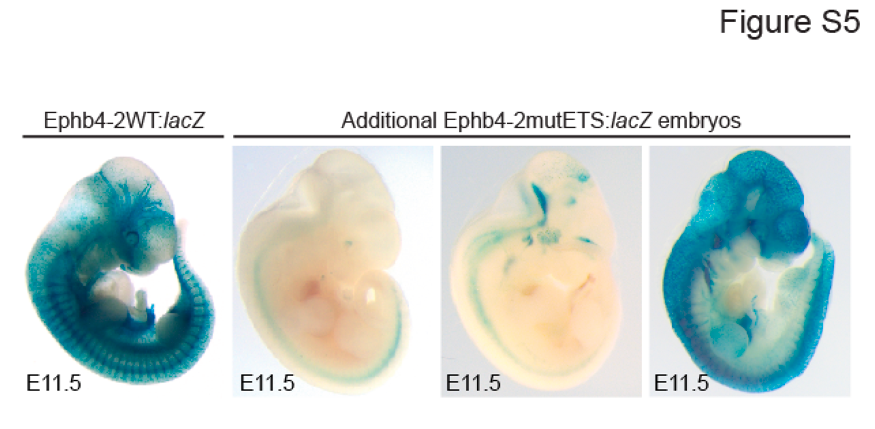
**

**Figure S5,** relating to Figure 2 in main text

**All additional F0 transgenic mouse embryos expressing Ephb4-2mutETS**

E11.5 independent F0 transgenic mouse embryos (all embryos not shown in Figure 2C), expressing either wild type (right panel) or ETS-motif mutated (left panels) versions of the Ephb4-2:*lacZ* transgene.


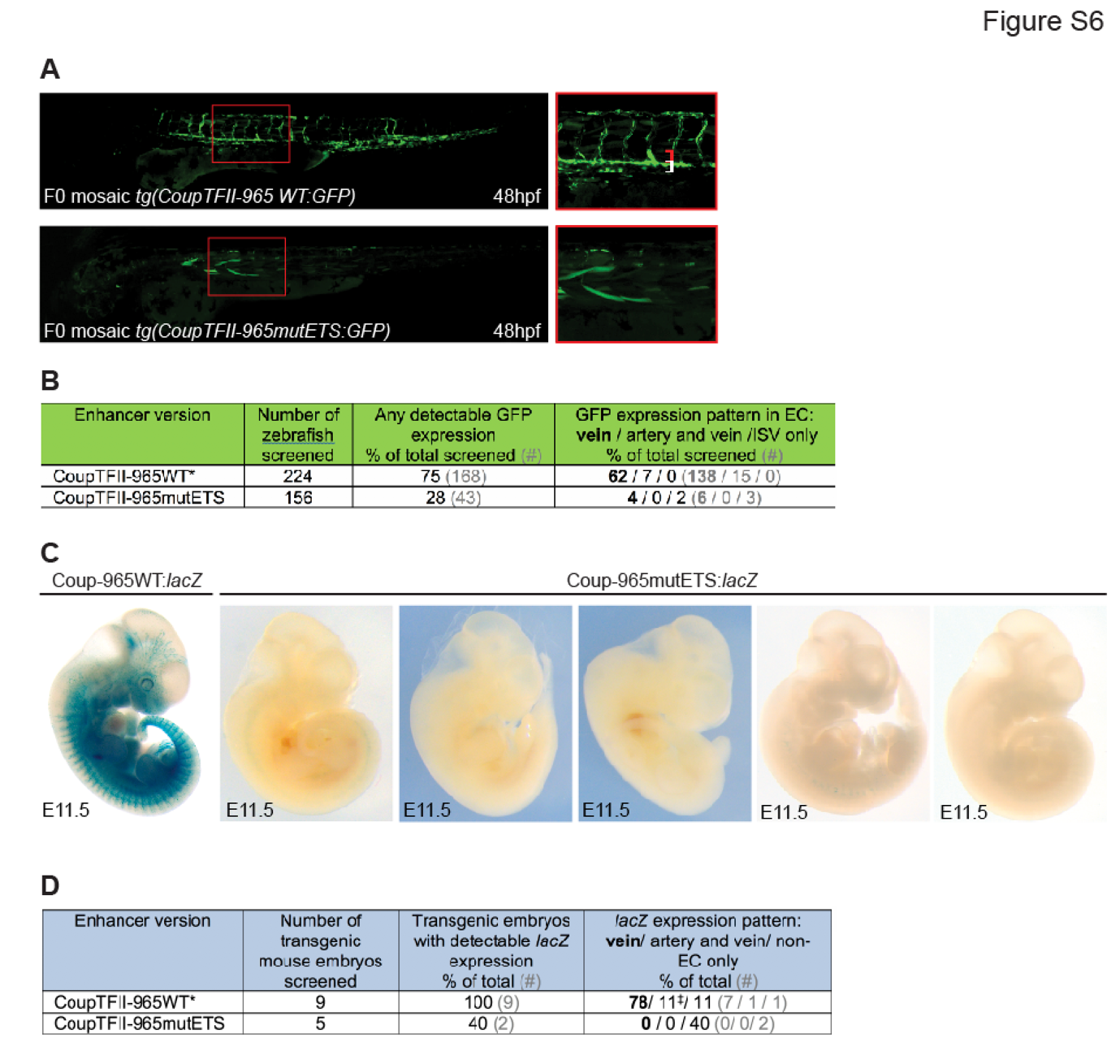


**Figure S6,** relating to Figure 2 in the main text.

**ETS factor motifs are required for venous CoupTFII-965 enhancer activity**

**A.** Representative 48hpf F0 Tol2-mediated mosaic transgenic zebrafish expressing either wild type (upper panel) or ETS-motif mutated (lower panel) versions of the CoupTFII-965:GFP transgene. Red box denotes region shown at high magnification on the left, red bracket indicates dorsal aorta, white bracket indicates cardinal vein. **B.** Table summarizing the n numbers and patterns of GFP expression in F0 Tol2-mediated transgenic zebrafish. * indicates transgenic zebrafish already reported in Neal et al., 2019. Note that the total numbers of zebrafish screened is lower than reported in Neal et al., 2019, as they exclude analysis that did not record vein/arterial/isv expression patterns. **C.** Representative E11.5 F0 transgenic mouse embryos expressing either wild type (left panel) or ETS-motif mutated (right panels) versions of CoupTFII:*lacZ* transgenes. cev = branches of cerebral venous plexus, cv = cardinal vein, isv = intersomitic vessel, nt = neural tube. All additional transgenic embryos are shown in Fig. S5. **D.** Table summarizing the n numbers and patterns of X-gal staining in F0 transgenic mouse embryos. * denotes data initially reported in Neal et al., 2019.


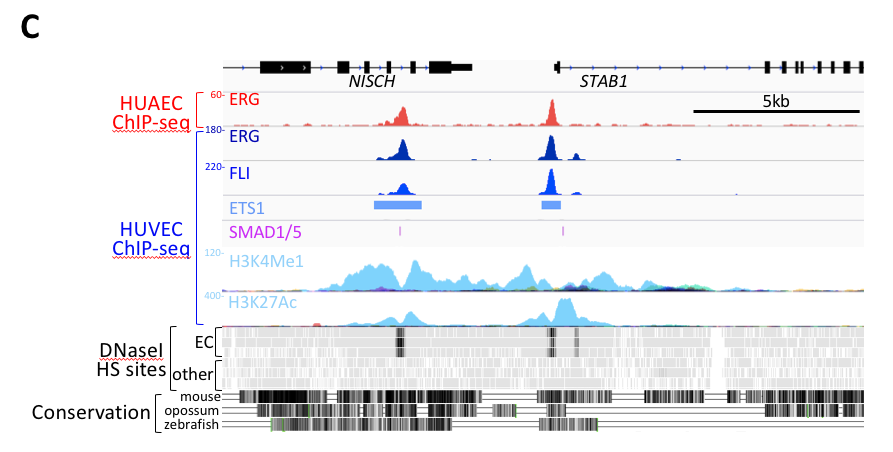

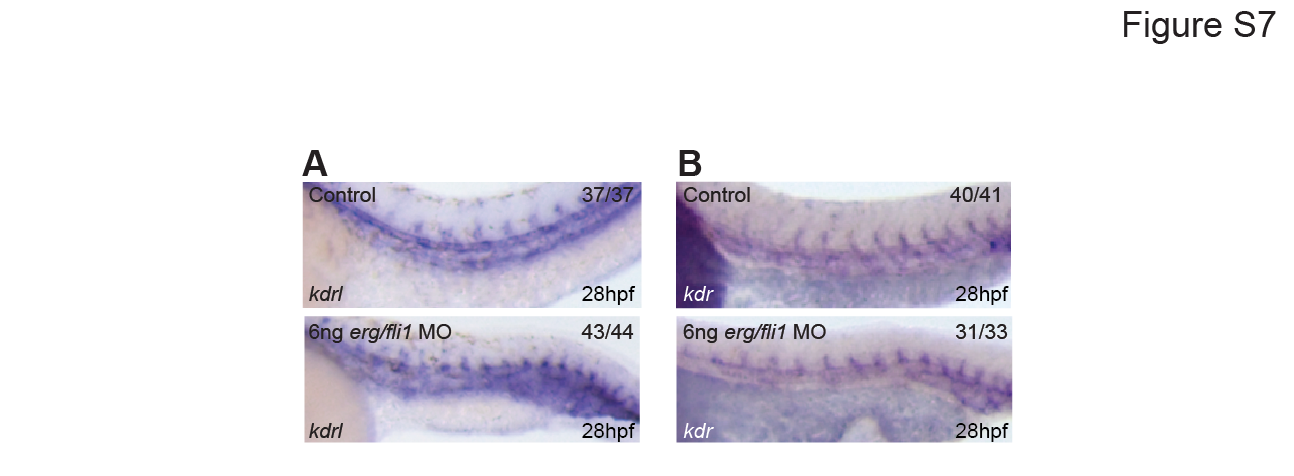


**Figure S7,** relating to Figure 3 in the main text.

**A-B.** Representative 28 hpf zebrafish after for VEGFA receptors *kdrl* (**A**) and *kdr* (**B**) demonstrates that morpholino-induced *erg/fli1* knockdown does not affect kdrl or kdr expression levels. Numbers on top right indicate number of embryos with the predominant and displayed phenotype per total number of embryos analysed.

**C**. IGV genome browser view the STAB1 loci indicating regions of ERG binding from (Sissaoui et al., 2020) (HUAEC in red, HUVEC in dark blue), FLI binding from (Nagai et al., 2018) (in HUVECs, royal blue) ETS1 binding from (Chen et al., 2017) (in HUVEC after 4 hours VEGFA, lighter blue horizontal line) and SMAD1/5 binding (Morikawa et al., 2011)(in HUVEC after BMP9 stimulation) aligned to UCSC genome browser view of enhancer histone marks (pale blue, in HUVECs), DNase I HS (black heat map, HUVECs and HMVECs) and sequence conservation between human and mouse, opossum and zebrafish.


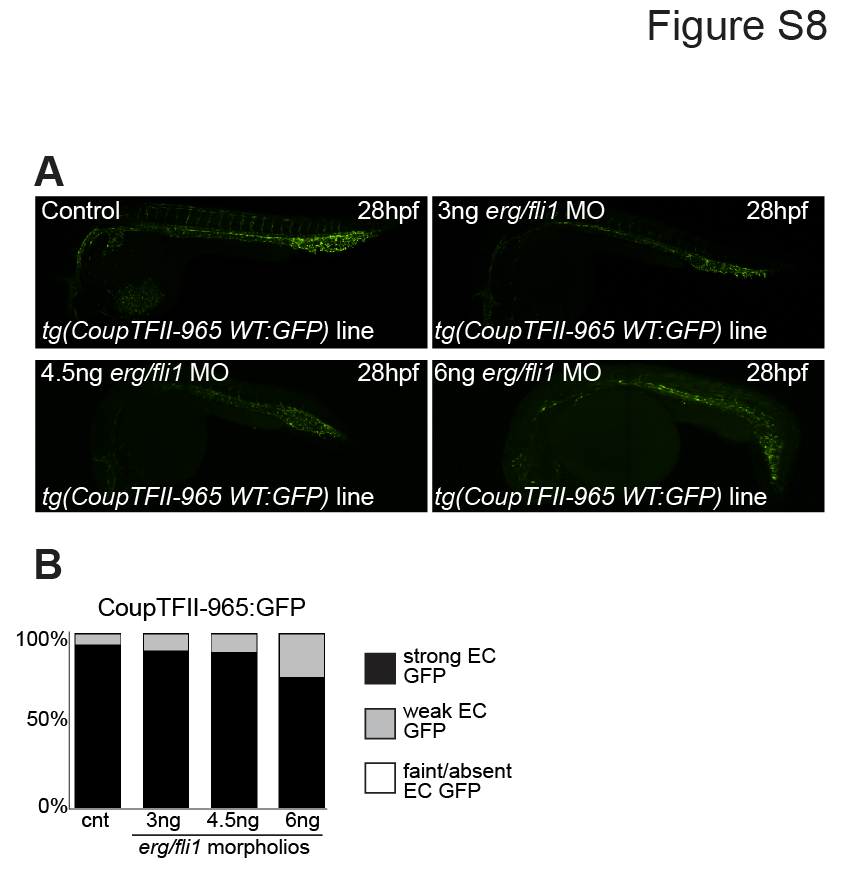


**Figure S8,** relating to Figure 3 in the main text.

**A.** Representative *tg(CoupTFII-965WT:GFP)* transgenic zebrafish after morpholino-induced *erg/fli1* knockdown. **B**. Graphs depicting observed GFP expression levels. CoupTFII-965:GFP cnt n=196, 3ng MO n=175, 4.5ng MO n=154, 6ng MO n=111.

**
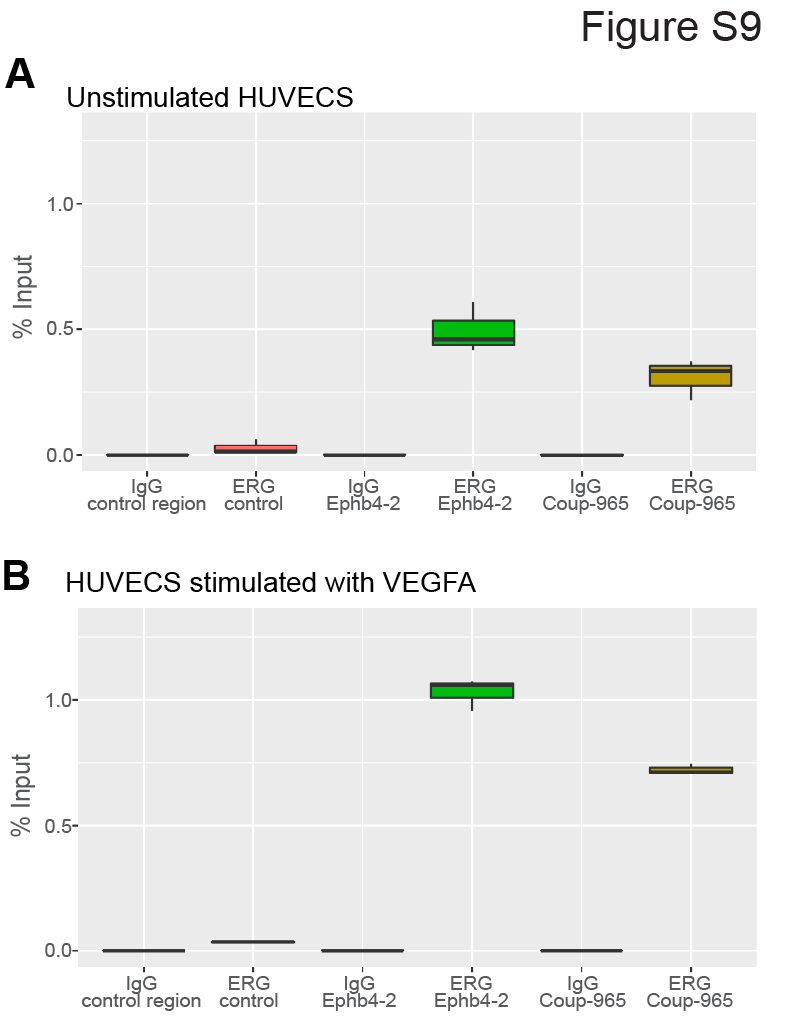
**

**Figure S9,** relating to Figure 4 in the main text.

**Further ChIP-qPCR results including IgG controls**

**A.** Box and whiskers plot of ChIP-qPCR data shows no significant enrichment of ERG1 binding compared to IgG control in unstimulated HUVECS at a control intergenic region (pink p>0.05) but did show significant ERG binding at the Ephb4-2 enhancer (green, p<0.001) and CoupTFII-965 enhancer (yellow, p<0.001).

**B**. Box and whiskers plot of ChIP-qPCR data shows no significant enrichment of ERG binding in VEGFA-stimulated HUVECS at the control intergenic region (pink p>0.05) but shows significant binding at the Ephb4-2 enhancer region (green, p<0.001) and the CoupTFII-965 enhancer region (yellow, p<0.001).

Horizontal lines = medians, boxes = interquartile range (IQR); vertical lines = minimal/maximal values.

**
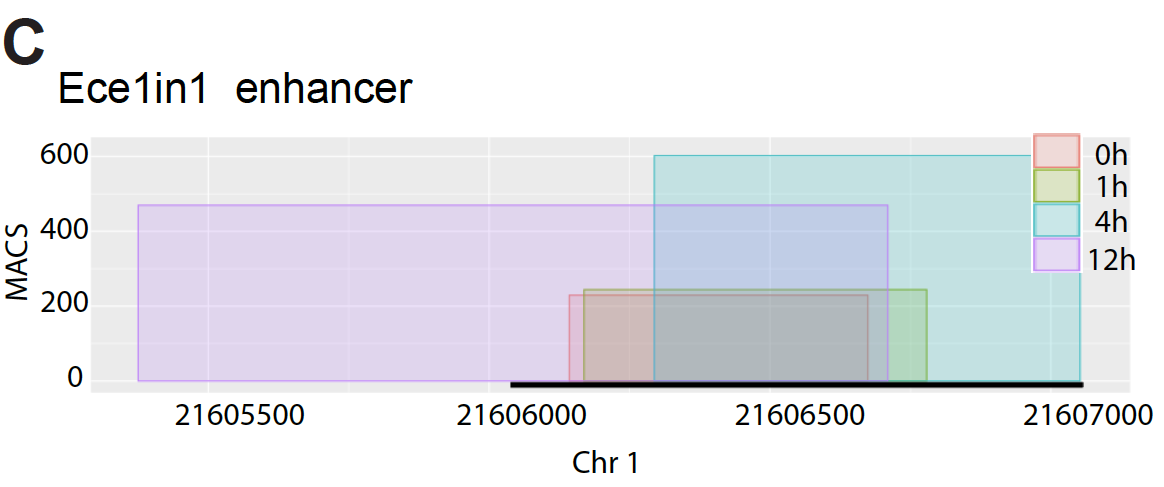

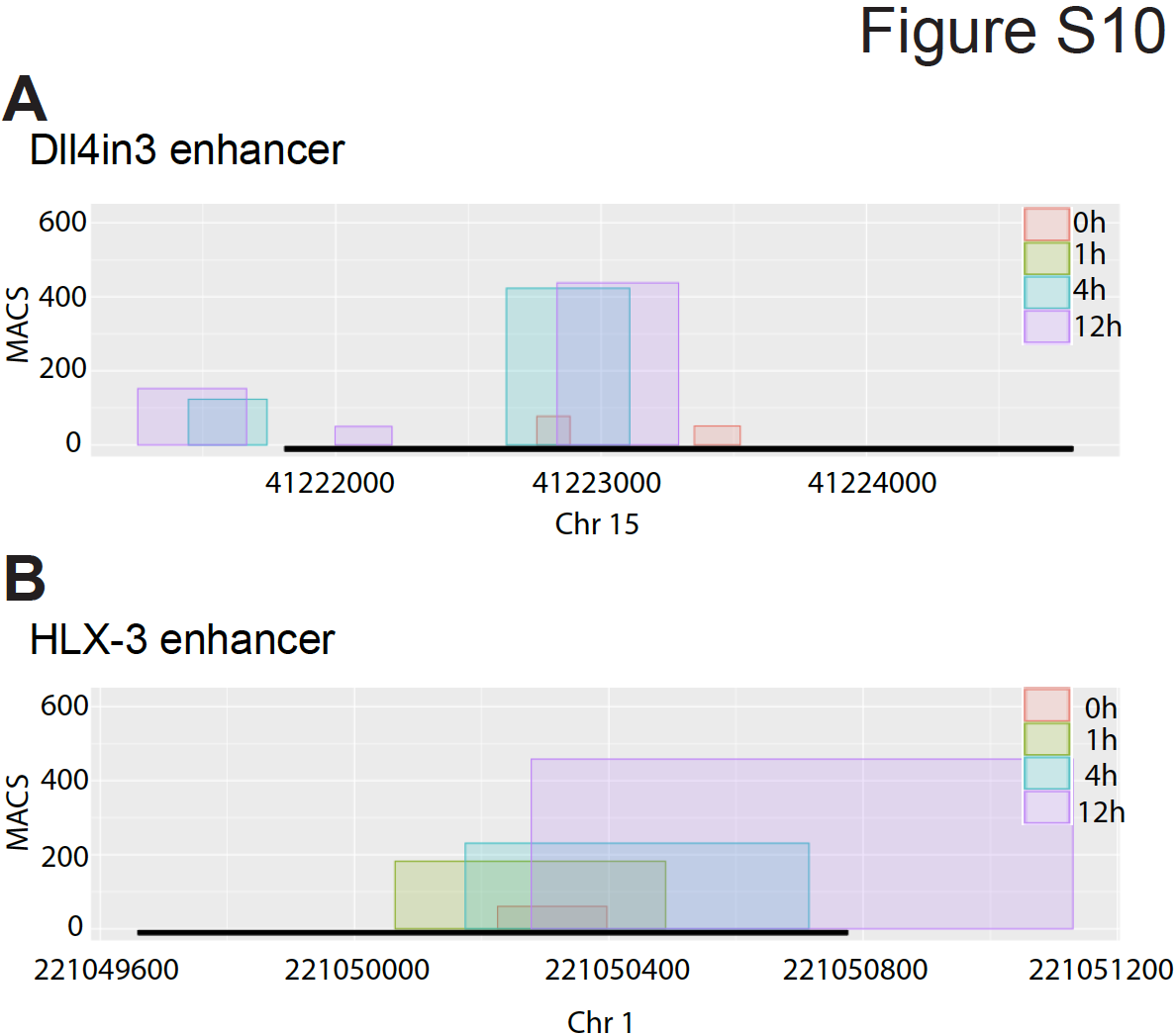
**

**Figure S10,** relating to Figure 4 in the main text.

**VEGFA signalling increases ETS binding to arterial and angiogenic enhancers.**

ETS1 binding at **(A)** the arterial/angiogenic Dll4in3, **(B)** the angiogenic HLX-3 and **(C)** the arterial Ece1intron1 (Robinson et al., 2014) enhancer regions. ETS1 binding is increased in the hours after VEGFA stimulation. Box width indicates region of ETS1 binding and box height indicates the maximal MACS score for this region after 0h (red), 1 hour (green), 4 hours (blue) and 12 hours of VEGFA stimulation. Black bar indicates orthologous enhancer region, x axis covers a 5kb genomic region. Numbers indicate chromosome location on hg19 build. Data reanalysed from ETS1 ChIP-seq by (Chen et al., 2017)


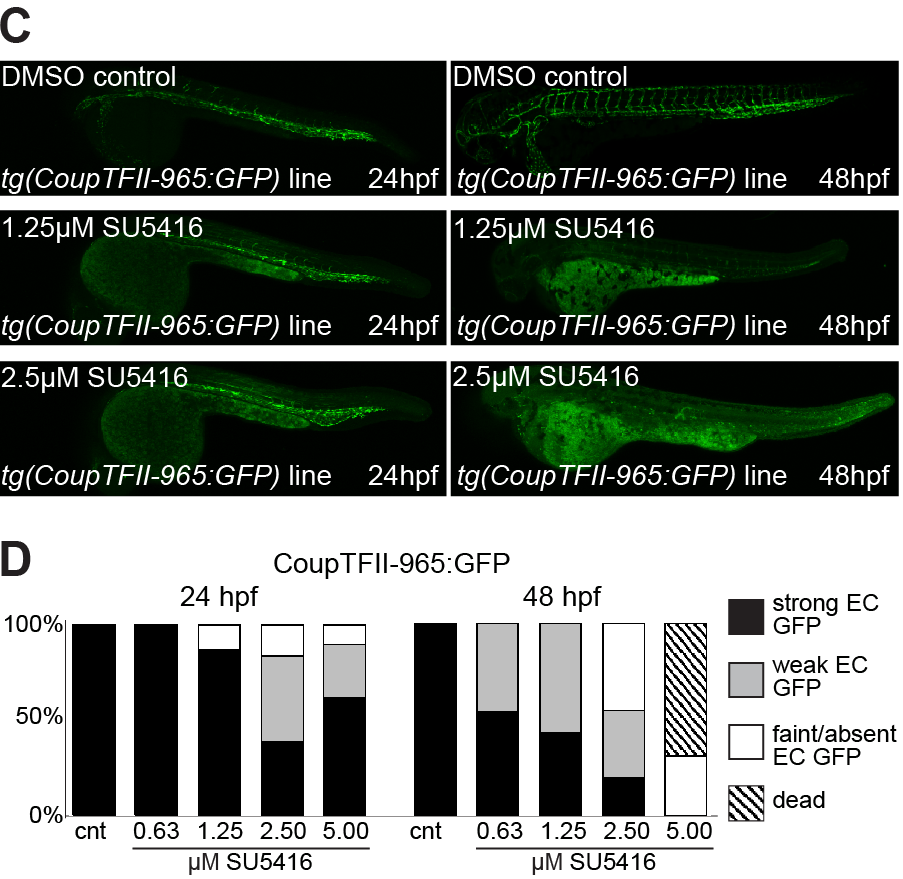

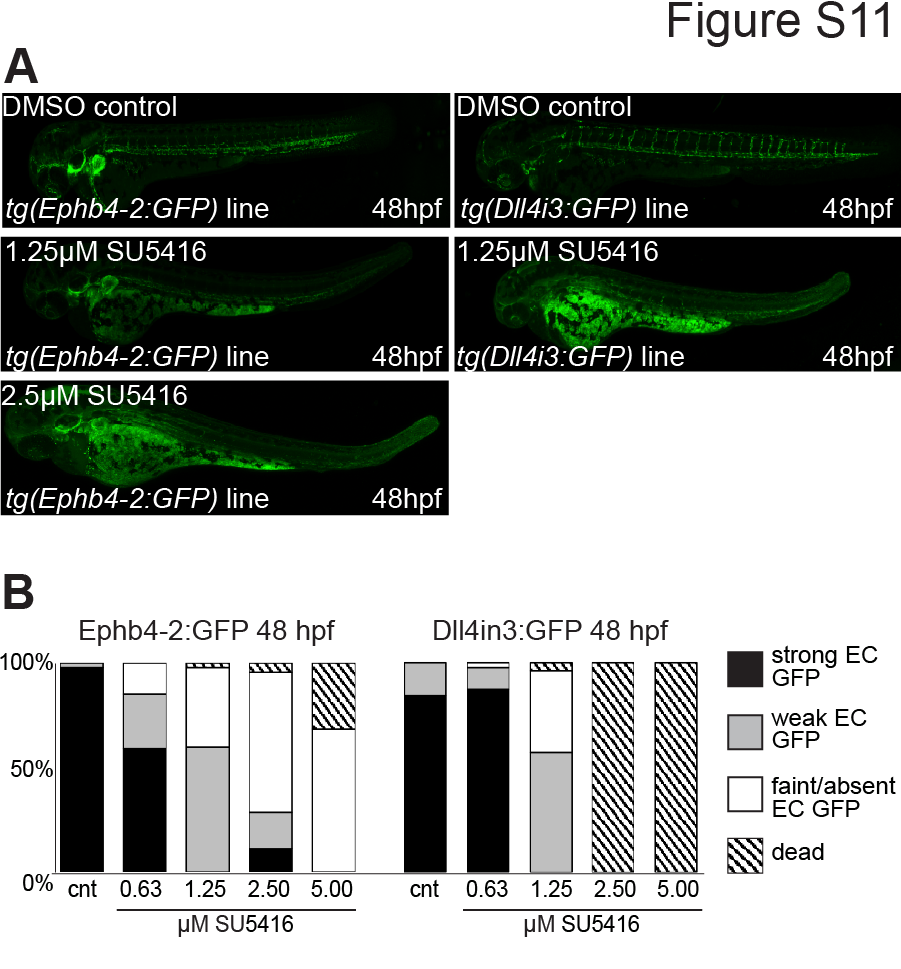


­­­­­­

**Figure S11**, relating to Figure 5 in the main text

**Further data supporting a role for VEGFA signalling in both venous and arterial enhancer activity**

**A**. Representative 48 hpf venous *tg(Ephb4-2:GFP)* and arterial/angiogenic *tg(Dll4in3:GFP)* zebrafish embryos treated with either DMSO control or different concentrations of VEGFR inhibitor SU5416. **B**. Graph depicting observed GFP expression levels in 48 hpf transgenic embryos treated with DMSO control or different levels of SU5416. Ephb4-2:GFP cnt n=117, 0.63µM SU5416 n=27, 1.25µM SU5416 n=50, 2.5µM SU5416 n=134, 5µM SU5416 n=38. Dll4in3:GFP cnt n=32, 0.63µM SU5416 n=55, 1.25µM SU5416 n=28, 2.5µM SU5416 n=26, 5µM SU5416 n=33. **C**. Representative 24 and 48 hpf venous *tg(CoupTFII-965:GFP)* zebrafish embryos treated with either DMSO control or different concentrations of VEGFR inhibitor SU5416. **D**. Graph depicting observed GFP expression levels in 24 and 48 hpf transgenic embryos treated with DMSO control or different levels of SU5416. CoupTFII-965:GFP at 24 hpf cnt n=38, 0.63µM SU5416 n=37, 1.25µM SU5416 n=37, 2.5µM SU5416 n=36, 5µM SU5416 n=39. CoupTFII-965:GFP at 48 hpf cnt n=148, 0.63µM SU5416 n=37, 1.25µM SU5416 n=37, 2.5µM SU5416 n=144, 5µM SU5416 n=35.


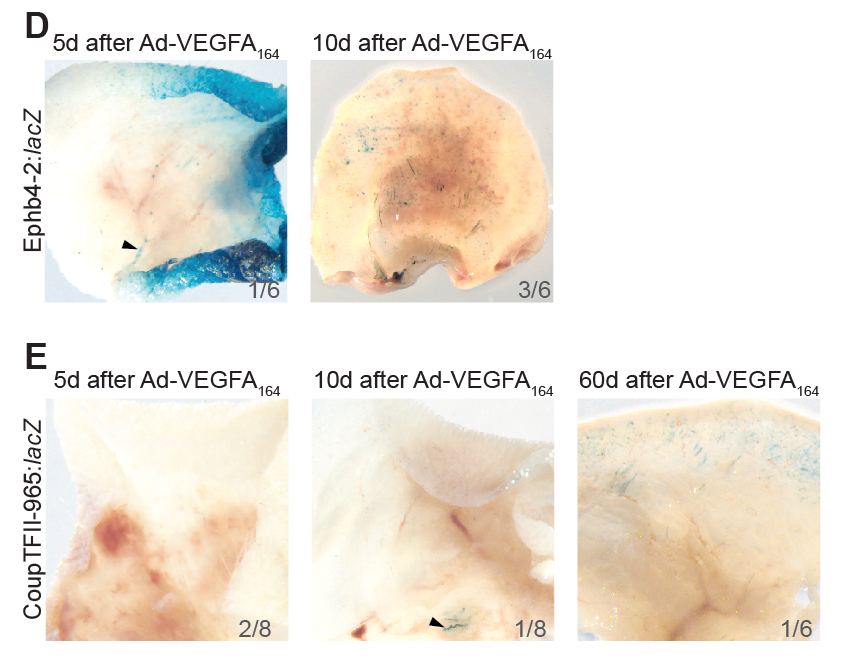


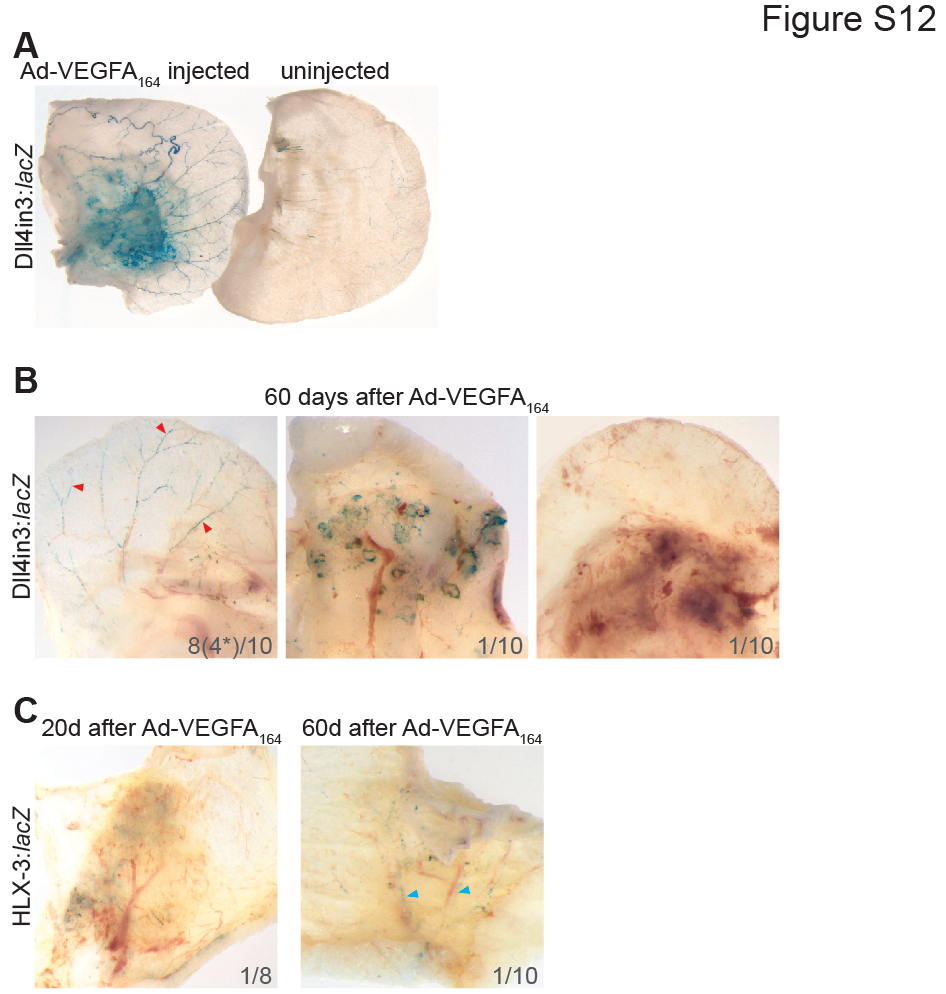
**Figure S12,** relating to Fig. 6 in main text.

**Additional data showing intradermal injection of Ad-VEGFA_164_ driving arterial and angiogenic enhancer activity.**

**A.** Dll4in3:*lacZ* staining in uninjected ears imaged together with an Ad-VEGFA_164_-injected ear. Both were stained in X-gal overnight. X-gal staining is much more intense in arteries of ears that were Ad-VEGF_164_ injected than uninjected controls.

**B-E**. Example of minority X-gal staining patterns seen after Ad-VEGFA_164_ injection in Dll4in3:*lacZ* (**B**), HLX-3:*lacZ* (**C**), Ephb4-2:*lacZ* (**D**) and CoupTFII-965:*lacZ* (**E**) ears. N numbers are indicated on images in bottom left corner, represented as number of ears similar to image shown/total number of ears investigated.

Red arrowhead=artery, blue arrowhead=vein, black arrowhead=blood vessel.

**
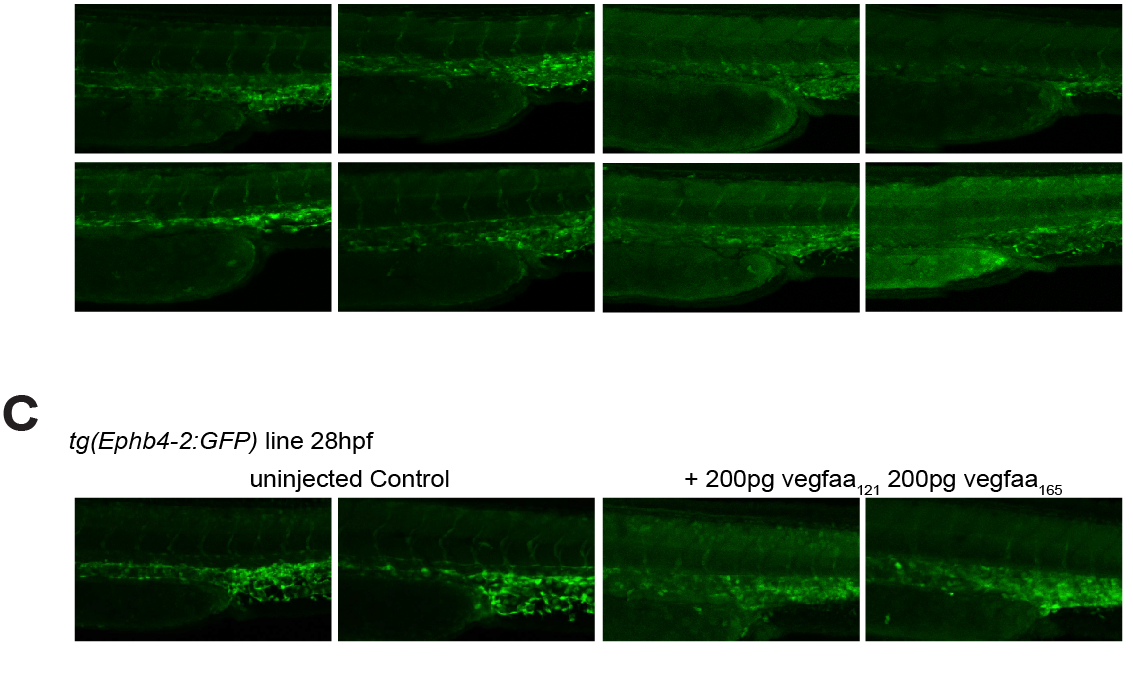

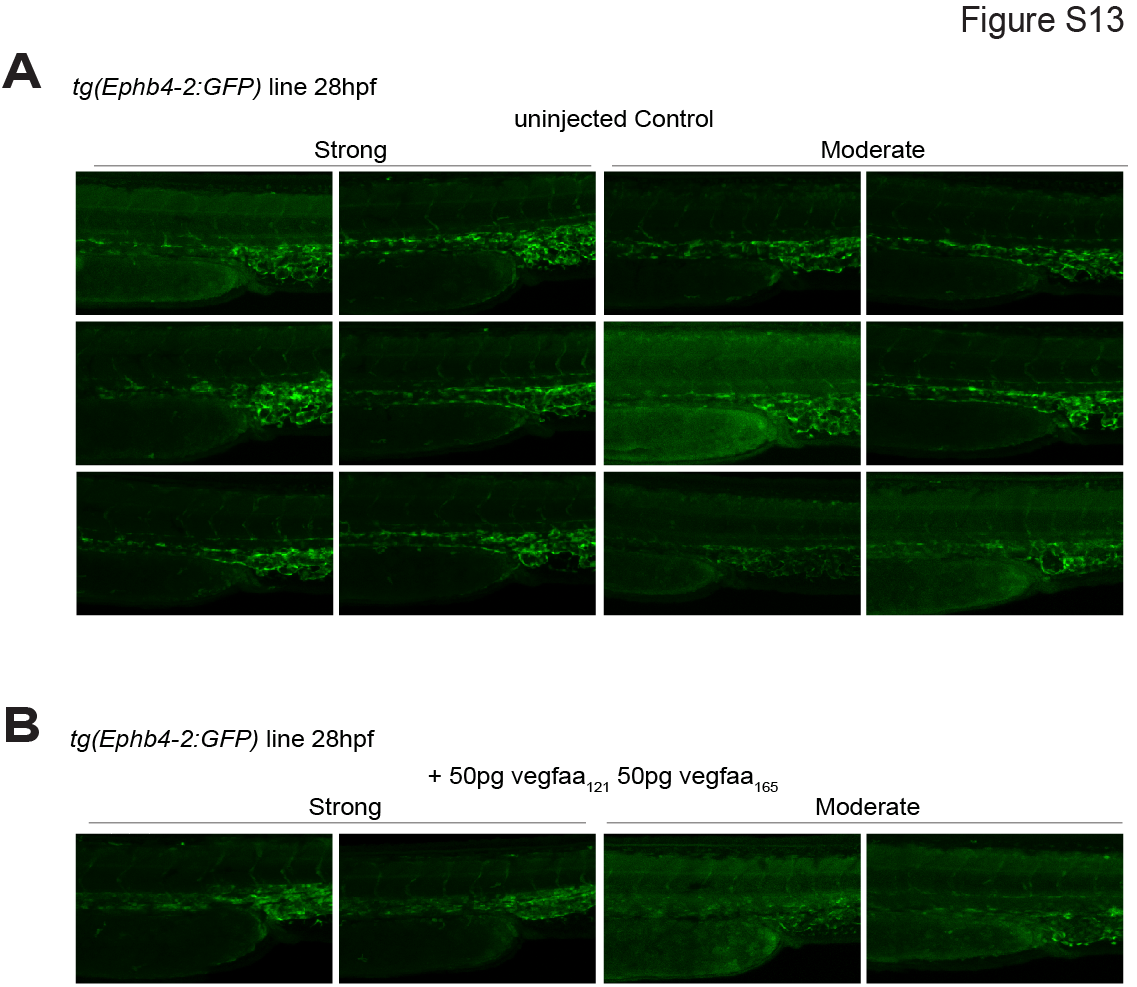
Figure S13,** relating to Fig. 7 in main text.

**More data demonstrating that VEGFA overexpression does not increase the intensity of venous Ephb4-2:GFP expression during embryonic development in zebrafish.**

**(A-B)** 12 representative 28 hpf control (**A**) or 50ng *vegfaa* mRNA injected (**B**) *tg(Ephb4-2:GFP)* transgenic embryos. Similar levels and patterns of GFP expression were seen in both groups, although some variability in GFP intensity was found with both WT and injected groups (represented here as either strong or moderate intensity levels). This is also graphically represented in Fig. 7.

**(C)** 2 representative 28 hpf control or 200ng *vegfaa* mRNA injected *tg(Ephb4-2:GFP)* transgenic embryos. Increased *vegfaa* levels did not result in a notable increase in GFP intensity (n=67 *vegfaa* injected, 71 control).


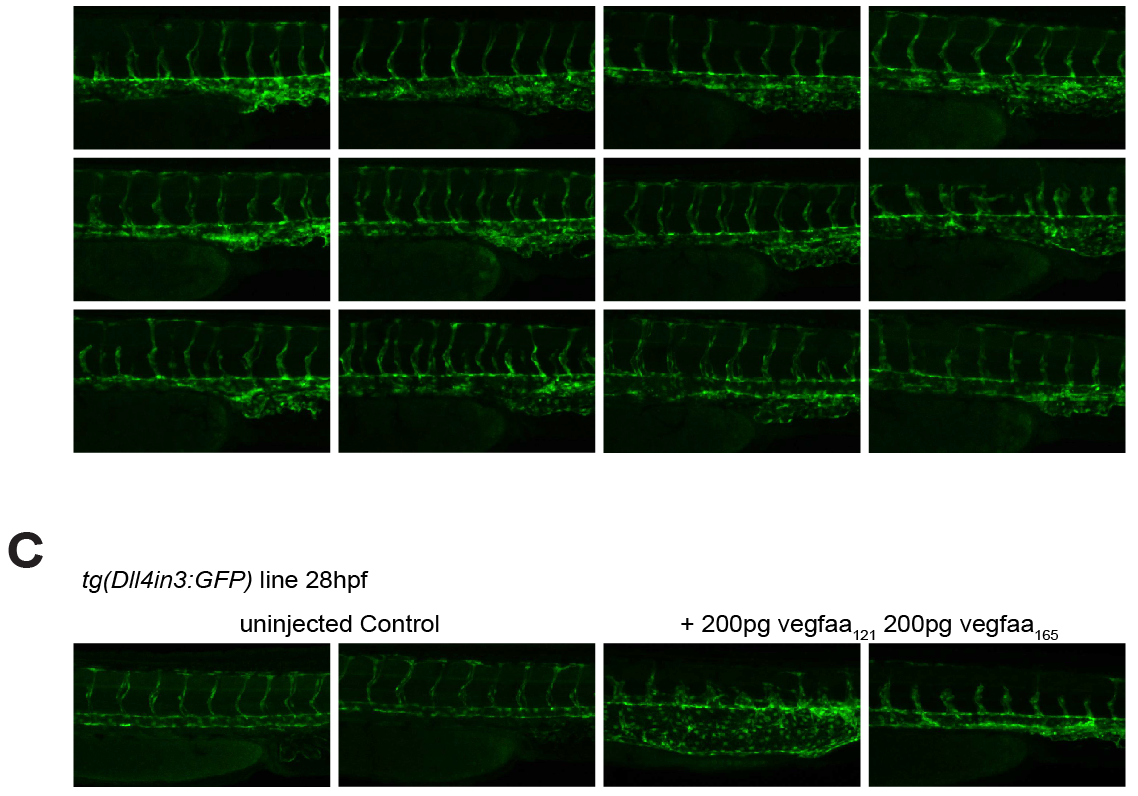

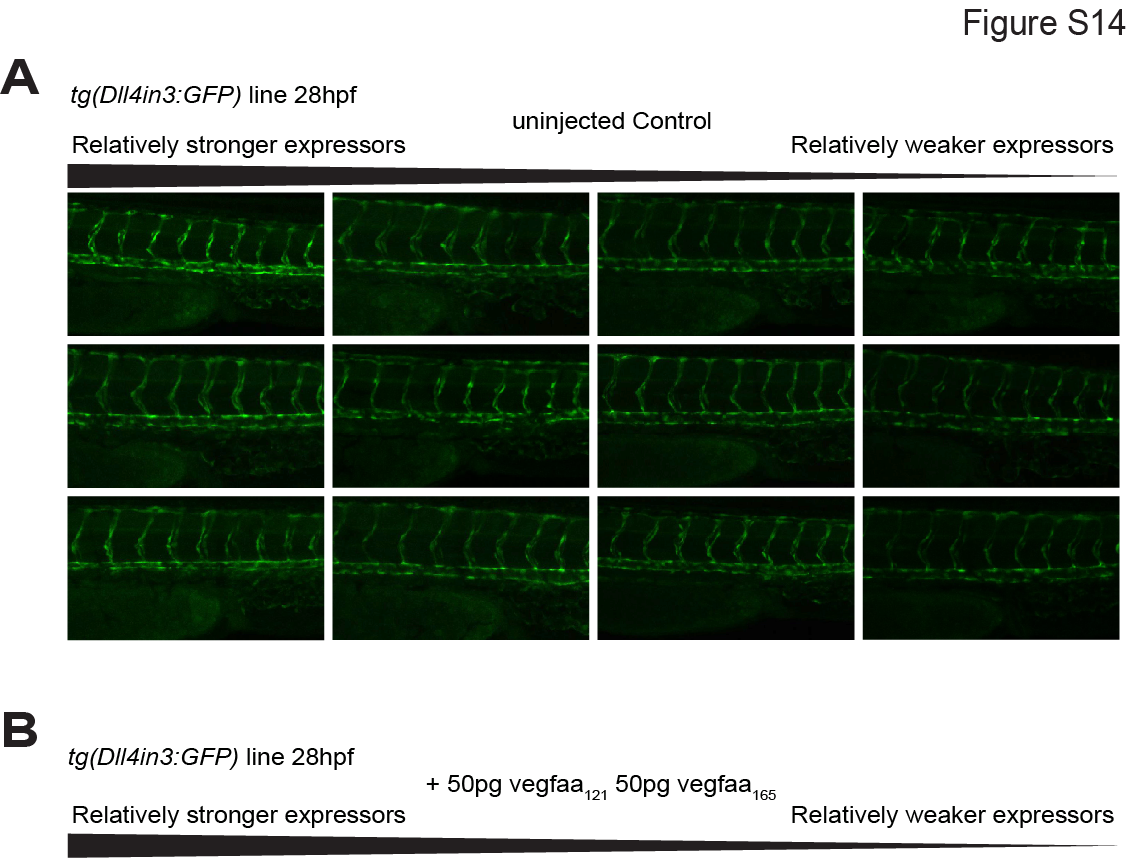


**Figure S14,** relating to Fig. 7 in main text

**VEGFA overexpression in arterial/angiogenic tg(Dll4in3:GFP) zebrafish**

**(A-B)** 12 representative 28 hpf control (**A**) or 50ng *vegfaa* mRNA injected (**B**) *tg(Dll4in3:GFP)* transgenic embryos. Similar levels and patterns of GFP expression were seen in both groups. Less variability was seen within the WT group comparative to tg(Ephb4-2:GFP) embryos, but the representative embryos are arranged according to GFP intensity levels for clarity.

**(C)** 2 representative 28 hpf control or 200ng *vegfaa* mRNA injected *tg(Dll4in3:GFP)* transgenic embryos. Increased *vegfaa* levels did not result in a notable increase in GFP intensity (n=51 *vegfaa* injected, 72 control).

**References for Supplemental Figures**

**Becker, P.W., Sacilotto, N., Nornes, S., Neal, A., Thomas, M.O., Liu, K., Preece, C., Ratnayaka, I., Davies, B., Bou-Gharios, G., De Val, S.** 2016. An Intronic Flk1 Enhancer Directs Arterial-Specific Expression via RBPJ-Mediated Venous Repression. *Arterioscler Thromb Vasc Biol* **36,** 1209–1219.

**Boutet, S.C., Quertermous, T., Fadel, B.M.,** 2001. Identification of an octamer element required for in vivo expression of the TIE1 gene in endothelial cells. *Biochem J* **360**, 23–29.

**Chen, J., Fu, Y., Day, D.S., Sun, Y., Wang, S., Liang, X., Gu, F., Zhang, F., Stevens, S.M., Zhou, P., Li, K., Zhang, Y., Lin, R.-Z., Smith, L.E.H., Zhang, J., Sun, K., Melero-Martin, J.M., Han, Z., Park, P.J., Zhang, B., Pu, W.T.,** 2017. VEGF amplifies transcription through ETS1 acetylation to enable angiogenesis. *Nat Commun* **8**, 383.

**Chiang, I.K.-N., Fritzsche, M., Pichol-Thievend, C., Neal, A., Holmes, K., Lagendijk, A., Overman, J., D'Angelo, D., Omini, A., Hermkens, D., Lesieur, E., Liu, K., Ratnayaka, I., Corada, M., Bou-Gharios, G., Carroll, J., Dejana, E., Schulte-Merker, S., Hogan, B., Beltrame, M., De Val, S., François, M.,** 2017. SoxF factors induce Notch1 expression via direct transcriptional regulation during early arterial development. *Development* **144**, 2629–2639.

**De Val, S., Chi, N.C., Meadows, S.M., Minovitsky, S., Anderson, J.P., Harris, I.S., Ehlers, M.L., Agarwal, P., Visel, A., Xu, S.-M., Pennacchio, L.A., Dubchak, I., Krieg, P.A., Stainier, D.Y.R., Black, B.L.,** 2008. Combinatorial Regulation of Endothelial Gene Expression by Ets and Forkhead Transcription Factors. *Cell* 135, 1053–1064.

**Göttgens, B., Broccardo, C., Sanchez, M.-J., Deveaux, S., Murphy, G., Göthert, J.R., Kotsopoulou, E., Kinston, S., Delaney, L., Piltz, S., Barton, L.M., Knezevic, K., Erber, W.N., Begley, C.G., Frampton, J., Green, A.R.,** 2004. The scl +18/19 stem cell enhancer is not required for hematopoiesis: identification of a 5' bifunctional hematopoietic-endothelial enhancer bound by Fli-1 and Elf-1. *Mol Cell Biol* **24**, 1870–1883.

**Kanki, Y., Kohro, T., Jiang, S., Tsutsumi, S., Mimura, I., Suehiro, J.-I., Wada, Y., Ohta, Y., Ihara, S., Iwanari, H., Naito, M., Hamakubo, T., Aburatani, H., Kodama, T., Minami, T.,** 2011. Epigenetically coordinated GATA2 binding is necessary for endothelium-specific endomucin expression. *The EMBO Journal* **30**, 2582–2595.

**Kappel, A., Rönicke, V., Damert, A., Flamme, I., Risau, W., Breier, G.,** 1999. Identification of vascular endothelial growth factor (VEGF) receptor-2 (Flk-1) promoter/enhancer sequences sufficient for angioblast and endothelial cell-specific transcription in transgenic mice. *Blood* **93**, 4284–4292.

**Khandekar, M., Brandt, W., Zhou, Y., Dagenais, S., Glover, T.W., Suzuki, N., Shimizu, R., Yamamoto, M., Lim, K.-C., Engel, J.D.,** 2007. A Gata2 intronic enhancer confers its pan-endothelia-specific regulation. *Development* **134**, 1703–1712.

**Morikawa, M., Koinuma, D., Tsutsumi, S., Vasilaki, E., Kanki, Y., Heldin, C.-H., Aburatani, H., Miyazono, K.,** 2011. ChIP-seq reveals cell type-specific binding patterns of BMP-specific Smads and a novel binding motif. *Nucleic Acids Research* **39**, 8712–8727.

**Nagai, N., Ohguchi, H., Nakaki, R., Matsumura, Y., Kanki, Y., Sakai, J., Aburatani, H., Minami, T.,** 2018. Downregulation of ERG and FLI1 expression in endothelial cells triggers endothelial-to-mesenchymal transition*. PLoS Genet* **14**, e1007826.

**Neal, A., Nornes, S., Payne, S., Wallace, M.D., Fritzsche, M., Louphrasitthiphol, P., Wilkinson, R.N., Chouliaras, K.M., Liu, K., Plant, K., Sholapurkar, R., Ratnayaka, I., Herzog, W., Bond, G., Chico, T., Bou-Gharios, G., De Val, S.,** 2019. Venous identity requires BMP signalling through ALK3. *Nat Commun* **10**, 453.

**Prandini, M.-H., Dreher, I., Bouillot, S., Benkerri, S., Moll, T., Huber, P.,** 2005. The human VE-cadherin promoter is subjected to organ-specific regulation and is activated in tumour angiogenesis. *Oncogene* **24**, 2992–3001.

**Robinson, A.S., Materna, S.C., Barnes, R.M., De Val, S., Xu, S.-M., Black, B.L.,** 2014. An arterial-specific enhancer of the human endothelin converting enzyme 1 (ECE1) gene is synergistically activated by Sox17, FoxC2, and Etv2. *Dev Biol* **395**, 379–389.

**Sacilotto, N., Chouliaras, K.M., Nikitenko, L.L., Lu, Y.W., Fritzsche, M., Wallace, M.D., Nornes, S., Garcia-Moreno, F., Payne, S., Bridges, E., Liu, K., Biggs, D., Ratnayaka, I., Herbert, S.P., Molnar, Z., Harris, A.L., Davies, B., Bond, G.L., Bou-Gharios, G., Schwarz, J.J., De Val, S.,** 2016. MEF2 transcription factors are key regulators of sprouting angiogenesis. *Genes Dev* **30**, 2297–2309.

**Sacilotto, N., Monteiro, R., Fritzsche, M., Becker, P.W., Sanchez-del-Campo, L., Liu, K., Pinheiro, P., Ratnayaka, I., Davies, B., Goding, C.R., Patient, R., Bou-Gharios, G., De Val, S.,** 2013. Analysis of Dll4 regulation reveals a combinatorial role for Sox and Notch in arterial development. *Proceedings of the National Academy of Sciences* **110**, 11893–11898.

**Sánchez, M., Göttgens, B., Sinclair, A.M., Stanley, M., Begley, C.G., Hunter, S., Green, A.R.,** 1999. An SCL 3' enhancer targets developing endothelium together with embryonic and adult haematopoietic progenitors. *Development* **126**, 3891–3904.

**Seki, T., Yun, J., Oh, S.P.,** 2003. Arterial endothelium-specific activin receptor-like kinase 1 expression suggests its role in arterialization and vascular remodeling. *Circ Res* **93**, 682–689.

**Sissaoui, S., Yu, J., Yan, A., Li, R., Yukselen, O., Kucukural, A., Zhu, L.J., Lawson, N.D.,** 2020. Genomic Characterization of Endothelial Enhancers Reveals a Multifunctional Role for NR2F2 in Regulation of Arteriovenous Gene Expression. *Circ Res* **126**, 875–888.

**Wu, J., Iwata, F., Grass, J.A., Osborne, C.S., Elnitski, L., Fraser, P., Ohneda, O., Yamamoto, M., Bresnick, E.H.,** 2005. Molecular determinants of NOTCH4 transcription in vascular endothelium. *Mol Cell Biol* **25**, 1458–1474.

**Zhou, P., Gu, F., Zhang, L., Akerberg, B.N., Ma, Q., Li, K., He, A., Lin, Z., Stevens, S.M., Bin Zhou, Pu, W.T.,** 2017. Mapping cell type-specific transcriptional enhancers using high affinity, lineage-specific Ep300 bioChIP-seq. *eLife Sciences* **6**, e22039.
